# Transcutaneous auricular vagus nerve stimulation during short‐term motor practice drives cortical plasticity without behavioural improvement

**DOI:** 10.1113/JP290398

**Published:** 2026-06-19

**Authors:** Kento Nakagawa, Rieko Osu

**Affiliations:** ^1^ Faculty of Human Sciences Waseda University Tokorozawa Saitama Japan; ^2^ Department of Sports and Health Management, Faculty of Business and Information Sciences Jobu University Isesaki Gunma Japan

**Keywords:** F‐wave, locus coeruleus, motor cortex, motor learning, neuromodulation, noradrenaline, vagus nerve stimulation

## Abstract

**Abstract:**

Transcutaneous auricular vagus nerve stimulation (taVNS) is emerging as a promising non‐invasive neuromodulation technique to augment neurorehabilitation; however, its mechanisms in humans remain poorly understood. Animal studies indicate that VNS during motor skill practice drives task‐specific plasticity in the primary motor cortex (M1), but direct evidence in humans is limited. Here, we demonstrate that taVNS paired with dexterous motor skill practice selectively enhances cortical plasticity without boosting motor performance during short‐term training. Thirty‐one healthy adults practiced a two‐ball rotation task for 15 min. Participants were randomised to receive taVNS to the left tragus or sham stimulation during practice. Motor performance, M1 representation (transcranial magnetic stimulation mapping) and spinal excitability (F‐wave) were assessed pre‐ and post‐practice, while pupil diameter was monitored during practice. Motor performance improved similarly in both groups, whereas cortical map expansion was significantly greater in the taVNS group than in the sham group. F‐wave amplitude increased in the sham group but not in the taVNS group, suggesting a relative shift in the expression of practice‐related plasticity toward cortical circuits when training was paired with taVNS. This pattern suggests that taVNS may influence the hierarchical expression of motor plasticity. Moreover, taVNS elicited pupil dilation during practice, consistent with noradrenergic engagement. These findings suggest that taVNS facilitates task‐specific cortical reorganization in humans independent of immediate behavioural improvement. By relating taVNS‐induced plasticity to noradrenergic engagement and revealing differential cortical and spinal responses, this study offers novel mechanistic insights into how pairing taVNS with motor training may establish a neural basis for motor recovery.

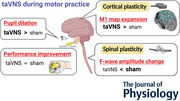

**Key points:**

- Previous research has shown that transcutaneous auricular vagus nerve stimulation (taVNS) is a promising neuromodulatory method, yet its effects on human motor learning and underlying plasticity mechanisms remain unclear.
- This study shows that, in healthy adults, taVNS paired with dexterous motor practice enhanced cortical plasticity (increased M1 map size) without immediate behavioural improvement.
- By contrast, sham stimulation alone increased F‐wave amplitude, suggesting that taVNS may promote a relative shift in the expression of plasticity toward cortical circuits.
- Pupillometric monitoring revealed enhanced noradrenergic activity during taVNS‑paired training, suggesting a neuromodulatory pathway associated with cortical plasticity.
- These findings highlight the potential of taVNS to modulate central plasticity mechanisms, offering a novel strategy that could be translated into rehabilitation interventions targeting neural recovery in neurological disorders.

## Graphical abstract

**Abstract figure legend** Transcutaneous auricular vagus nerve stimulation (taVNS) was applied during short‐term dexterous motor practice to examine its effects on behavioural performance and neural plasticity. Healthy participants practiced a two‐ball rotation task while receiving taVNS or sham stimulation. Motor performance improved similarly in both groups. However, taVNS induced greater expansion of the primary motor cortex (M1) representation, indicating enhanced cortical plasticity. In contrast, F‐wave amplitude increased in the sham group but not in the taVNS group, suggesting a relatively smaller change in spinal excitability with taVNS. Pupillometry revealed greater pupil dilation during practice in the taVNS group, consistent with enhanced noradrenergic engagement. These findings suggest that taVNS during dexterous motor practice preferentially facilitates cortical plasticity without immediate behavioural gain and may shift the expression of plasticity toward supraspinal mechanisms.

## Introduction

Enhancing neuroplasticity is a key strategy for promoting neurorehabilitation and motor skill acquisition. Vagus nerve stimulation (VNS) has shown promise in enhancing motor recovery, supported by both animal models and clinical trials in stroke patients (de Melo et al., 2023; Gerges et al., 2024). Transcutaneous auricular VNS (taVNS) provides a non‐invasive and safe alternative to invasive VNS and has therefore attracted growing therapeutic interest. Recent meta‐analyses demonstrated the safety of taVNS (Kim et al., 2022) and superior therapeutic effects compared to other neuromodulation techniques in stroke patients (Ahmed et al., 2023). However, the underlying therapeutic mechanisms of taVNS remain unclear.

No study has yet shown an improvement in dexterous motor skills in able‐bodied individuals through the use of taVNS. Animal studies have shown that invasive VNS delivered during dexterous motor training induces plastic changes in the primary motor cortex (M1) representation in a task‐dependent manner (Morrison et al., 2019; Porter et al., 2012). Therefore, pairing taVNS with motor skill training may facilitate plastic changes in motor‐related neural circuits innervating task‐relevant muscles. In humans, studies examining the effects of taVNS in the absence of a motor task on M1 excitability or inhibition have yielded inconsistent results (Gerges et al., 2025; Mertens et al., 2022; Yun et al., 2025). Notably, no study has yet assessed plastic changes in M1 map representation following taVNS. As M1 neurons project to spinal motoneurons via corticospinal pathways, cortical activation or even subthreshold plastic changes in M1 may also influence spinal excitability. Indeed, non‐invasive stimulation over M1 has been shown to modulate not only cortical excitability but also spinal circuits (Klomjai et al., 2022; Kondo et al., 2013). Voluntary motor execution relies on regulating spinal motoneuron excitability; hence, taVNS delivered during motor learning may influence both cortical and spinal motoneurons. However, the impact of taVNS on spinal motoneuron excitability remains unexplored. To isolate potential changes at the level of the motoneuron pool with minimal contributions from afferent pathways or descending corticospinal drive, we assessed F‐waves as an index of spinal motoneuron excitability. F‐waves primarily reflect motoneuron excitability through antidromic activation and backfiring of α‐motoneurons (Fisher, 1992). To our knowledge, the impact of taVNS on spinal motoneuron excitability has not been systematically examined. Furthermore, this approach allowed us to examine plastic changes at both cortical and spinal levels within the same experimental framework.

taVNS has also been shown to modulate autonomic and arousal systems. Studies have suggested that taVNS at rest activates the locus coeruleus–noradrenaline (LC‐NA) system, as reflected by pupil dilation, which has been widely used as a non‐invasive proxy for LC‐NA activity (Pervaz et al., 2025; Sharon et al., 2021). However, most evidence linking taVNS to LC‐NA activation has been obtained under resting conditions. Because engagement in motor behaviour itself modulates arousal and noradrenergic activity (Aston‐Jones & Cohen, 2005), it remains unclear whether taVNS delivered during ongoing motor behaviour engages similar neuromodulatory mechanisms. Monitoring pupil dynamics during taVNS paired with dexterous motor learning may therefore provide a useful window into how vagal stimulation interacts with task‐related noradrenergic modulation during motor behaviour.

Previous work in both animals and humans has highlighted the potential importance of stimulation timing. In several studies, phasic VNS was delivered concurrently with discrete movements such as reaching, grasping and lever pressing (Dawson et al., 2021; Khodaparast et al., 2014; Porter et al., 2012). An animal study further suggested enhanced motor improvements and selective modulation of M1 neurons when VNS is delivered immediately after successful movements (Bowles et al., 2022). Other rehabilitation studies reported beneficial effects of taVNS regardless of precise timing, for example when continuous taVNS at rest was combined with subsequent therapy (Li et al., 2022; Wu et al., 2020). Thus, the importance of stimulation timing remains unresolved. Furthermore, most studies have employed discrete tasks, leaving unclear whether taVNS can influence the learning of periodic motor skills. To address this gap, we employed periodic two‐ball rotation tasks, which require sequential and coordinated finger movements and have been widely used in motor learning paradigms (Hamada et al., 2023; Uehara et al., 2011). The aim of this skill is to rotate the balls faster, but even slow rotations can be regarded as a successful movement. Continuous taVNS during practice of two‐ball rotation can be regarded as stimulation delivered concurrently with ongoing successful movements, similar to phase‐specific VNS paired with discrete successes in prior studies.

We investigated the effects of taVNS paired with periodic dexterous motor skill practice on behavioural performance and plasticity in motor‐related neural circuits. We hypothesised that taVNS during motor practice facilitates motor learning, expands M1 representations of task‐relevant muscles and increases spinal motoneuron excitability, potentially through LC‐NA system engagement.

## Methods

### Ethical approval

This study was approved by the Human Research Ethics Committee of Waseda University (approval number: 2020–93). Written informed consent was obtained from all participants. The study conformed to the standards set by the *Declaration of Helsinki*, except for registration in a database because the study was not a clinical trial.

### Participants

Thirty‐one healthy right‐handed adults participated in this experiment. Participants were pseudo‐randomly assigned to either the taVNS group (*n* = 16; 23 ± 4 years; 3 females and 13 males) or the sham group (*n* = 15; 22 ± 5 years; 4 females and 11 males). Handedness was confirmed using the Edinburgh Handedness Inventory (Oldfield, 1971), with laterality quotient ranging from 68.4 to 100 (mean ± SD: 93.7 ± 10.2). None of the participants had prior experience with the two‐ball rotation task.

### Overall design and procedure

This study employed a randomised, single‐blind, sham‐controlled design. Participants in both groups were told that electrical stimulation would be delivered to the ear electrodes, but that the current might be too weak to perceive. Thus, all participants believed they were receiving real stimulation, regardless of group assignment. Since the stimulator did not include a sham mode and the experimenter manually adjusted the stimulation intensity, the experimenter was not blinded.

**Figure 1:**
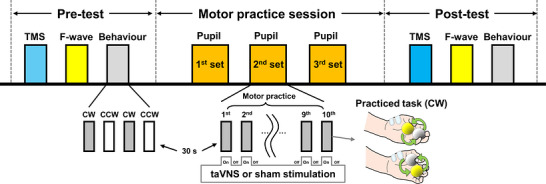
Experimental protocol In the Pre‐ and Post‐test, TMS mapping, F‐wave measurement and behavioural assessments were conducted. Behavioural tests consisted of 30‐s trials of clockwise (CW) and counterclockwise (CCW) ball rotation. During the practice session, participants performed 30 trials of 30‐s CW rotation with 30‐s rest intervals. taVNS or sham stimulation was applied during practice.

Figure 1 summarises the experimental protocol. The procedure was identical for both groups except for the presence (taVNS group) or absence (sham group) of electrical stimulation on the ear electrode during motor practice. Participants performed a two‐ball rotation task with the left hand, consisting of 30 practice trials (10 trials × 3 sets). During practice, taVNS or sham stimulation was applied to the tragus. Behavioural performance and neurophysiological assessments were conducted immediately before and after practice sessions (Pre‐ and Post‐tests) without taVNS electrodes attached. Each experimental session lasted approximately 3 h.

### taVNS

**Figure 2:**
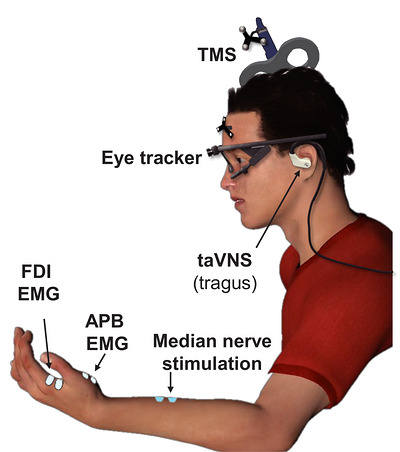
Experimental set‐up The human figure was computer‐generated using Poser 12 software and does not depict any real person. The equipment shown was created by the authors based on the actual apparatus used in the experiment.

To target the afferent auricular branch of the vagus nerve, a stimulation electrode (RELIfit‐Tragus, Soterix Medical Inc., Woodbridge, NJ, USA) was secured by hooking the device over the left ear, with the tip of the electrode positioned on the tragus (Fig. 2). The position was adjusted to maximise perceptual intensity and fixed with medical tape. The skin around the tragus was cleaned with alcohol wipes before placement. The tragus was chosen because it has been suggested as a suitable site for vagal modulation (Butt et al., 2020).

Stimulation was delivered using a dedicated device (0125tA, Soterix Medical Inc.) with parameters based on previous studies (Chang et al., 2021; Li et al., 2022; Wu et al., 2020): 30 Hz frequency, 300 µs pulse width, and a total duration of 900 s (30 blocks of 30‐s on and 30‐s off). Current intensity was set at the individual pain threshold or 0.5 mA below, because the device allowed only 0.5 mA increments. If stimulation at the pain threshold was reported as ‘intolerable’, intensity was reduced by 0.5 mA, which still exceeded the perception threshold in all participants. The mean stimulation intensity was 2.37 ± 1.41 mA. For sham stimulation, some previous studies applied current to the ear lobe (van Midden et al., 2023; Wu et al., 2020), where the vagal innervation is absent. However, as ear lobe stimulation may still influence motor‐related brain functions (van Midden et al., 2023), we used a no‐current sham condition with the electrode placed at the same tragus position, consistent with other studies (Dawson et al., 2021; Li et al., 2022). Therefore, no intensity adjustment was performed in the sham group. No participants reported adverse effects such as headaches.

### Motor task and practice

**Figure 3:**
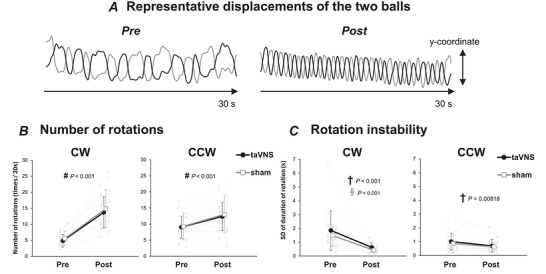
Behavioural results *A*, representative *y*‐axis displacement of the two balls (black: white ball; grey: yellow ball) digitised with DeepLabCut during 30‐s clockwise (CW) rotation in Pre‐ and Post‐tests. *B*, group data for the number of rotations (Pre *vs*. Post) in each group (taVNS, sham). Both groups improved in practiced CW and non‐practiced counterclockwise (CCW) rotations, with no significant group differences. *C*, group data for rotation instability (SD of cycle duration). In CW, instability decreased in both groups; in CCW, only the taVNS group showed a decrease. In the Pre–Post panels, small symbols represent individual participants (circles: taVNS; squares: sham), with lines connecting the Pre and Post values within each participant. Large symbols represent group means. Error bars: ±1 SD. #Significant main effect of time; †significant Pre–Post difference in taVNS group; §significant Pre–Post difference in sham group.

Participants practiced a periodic two‐ball rotation task with their left hand (Fig. 3*A*). Seated at a desk, they rotated two golf balls (33 mm in diameter, 45 g each; D1 Plus, HONMA Golf, Tokyo, Japan) placed in the supine left palm as quickly as possible in a clockwise direction. The balls were coloured yellow and white to facilitate tracking during later video analysis. Participants were instructed to maintain their gaze so that both the left hand and the monitor displaying task instructions were within view. If a ball was dropped, participants were instructed to pick it up immediately and continue the task.

During the pre‐ and post‐test, participants performed two 30‐s trials of the practiced clockwise (CW) rotation and two 30‐s trials of the unpracticed counterclockwise (CCW) rotation, each performed as fast as possible. Regardless of test or practice, every 30‐s motor task was preceded and followed by a 30‐s rest period without practice or stimulation. One practice set consisted of 10 alternating trials of 30‐s practice and 30‐s rest. Participants completed three sets in total, with breaks between sets determined individually (typically 1–2 min). Task instructions, trial timing and elapsed time were displayed on the monitor to ensure standardised practice across participants.

For behavioural assessment, performance was recorded using a webcam (D600, EMEET, Shenzhen, China) mounted on a tripod (Q111H, Say Good) above the participant's hand, at a frame rate of 60 fps. Video data were analysed using DeepLabCut, an open source markerless pose estimation toolbox with deep neural networks (Mathis et al., 2018). The *y*‐axis displacement of one tracked ball was used to compute two parameters in MATLAB (MathWorks, Natick, MA, USA): (1) the number of rotations, determined by peak cycle counts, and (2) rotation instability, quantified as the standard deviation of cycle length.

### Transcranial magnetic stimulation measurement

Electromyography (EMG) signals were recorded from the left abductor pollicis brevis (APB) and first dorsal interosseous muscle (FDI) using bipolar Ag/AgCl surface electrodes (Vitrode F‐150S, 18 × 36 mm, Nihon Kohden, Tokyo, Japan) (Fig. 2). EMG signals were amplified (MEB‐6108 amplifier, Nihon Kohden), band‐pass filtered (5–1500 Hz), and digitised at 4000 Hz with an A/D converter (Powerlab, ADInstrument, Sydney, Australia) for both motor evoked potential (MEP) and F‐wave recordings. Single‐pulse monophasic transcranial magnetic stimulation (TMS) was delivered with a Magstim 200 (Magstim, Whitland, UK) through a 70‐mm figure‐of‐eight coil. Participants were seated comfortably and instructed to remain relaxed throughout the measurements.

The coil was positioned over the site that elicited the largest MEP in the APB, which was designated as the hotspot. Resting motor threshold (RMT) was defined as the lowest TMS intensity that evoked MEPs >0.05 mV in at least 5 of 10 trials in the APB.

**Figure 4:**
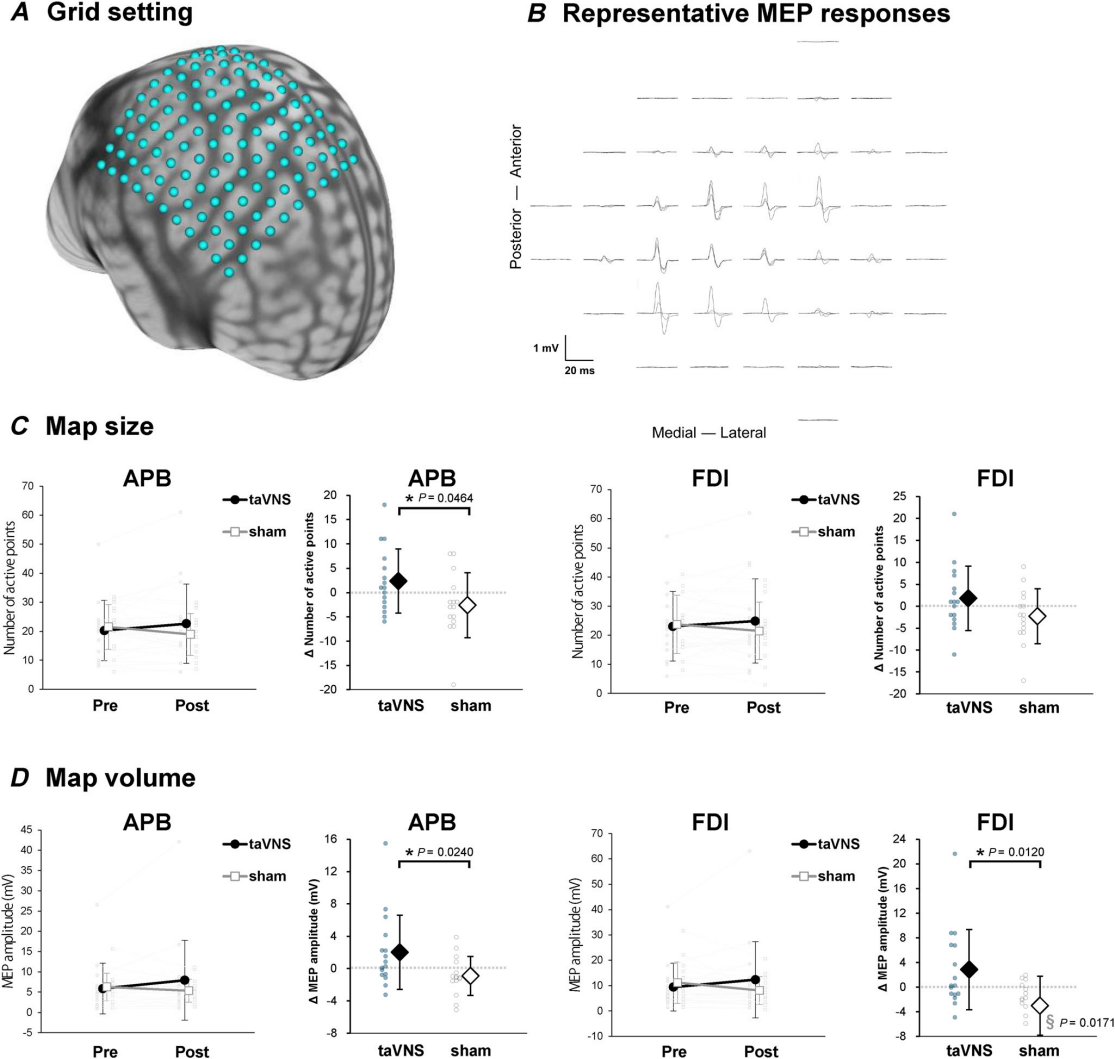
TMS motor mapping and results *A*, 11 × 11 grid centred on APB hotspot. *B*, representative APB MEPs from one participant in the Pre‐test (3 traces per grid point). *C*, group data for the map size (number of active points). *D*, group data for the map volume (sum of mean MEP amplitudes at active points). In the Pre–Post panels, small symbols represent individual participants (circles: taVNS; squares: sham), with lines connecting the Pre and Post values within each participant. Large symbols represent group means. Error bars indicate ±SD. In the Δ panels, each dot represents the individual change (Post−Pre), diamonds represent group means and error bars indicate ±SD. The horizontal dashed line indicates zero change. *taVNS *versus* sham; §Δ *versus* zero.

Motor maps were then obtained using TMS at 120% RMT (taVNS: 61.3 ± 10.4% of maximal stimulator output (MSO), sham: 58.9 ± 11.9% MSO) within an 11 × 11 cm grid (1 cm spacing) centred on the hotspot (Fig. 4*A*). Coil position was continuously monitored with a navigation system (Brainsight version 2.2.8, Rogue Research, Montreal, Canada). Coil orientation was held constant relative to the hotspot, while tilt was adjusted to maintain tangential contact with the scalp. Three TMSs were delivered at each grid point every 3–4 s, guided by the navigation system. This number of stimuli was chosen to minimise the duration of post‐intervention assessment given the uncertain temporal stability of the induced plasticity. The validity of using three stimuli per grid point was assessed by comparing mapping outcomes obtained using three *versus* five stimuli per grid point (see Results). Mapping began at the hotspot and expanded outward. The following stimulating site was randomly chosen from one of the four neighbours of active points (mean APB MEP amplitude >0.05 mV) that had not yet been tested. Sites with mean APB MEP <0.05 mV were defined as inactive. Mapping continued until the entire map was surrounded by inactive sites. The same procedure was applied during both pre‐ and post‐testing sessions. All TMS measurements were performed by the same experimenter.

**Figure 5:**
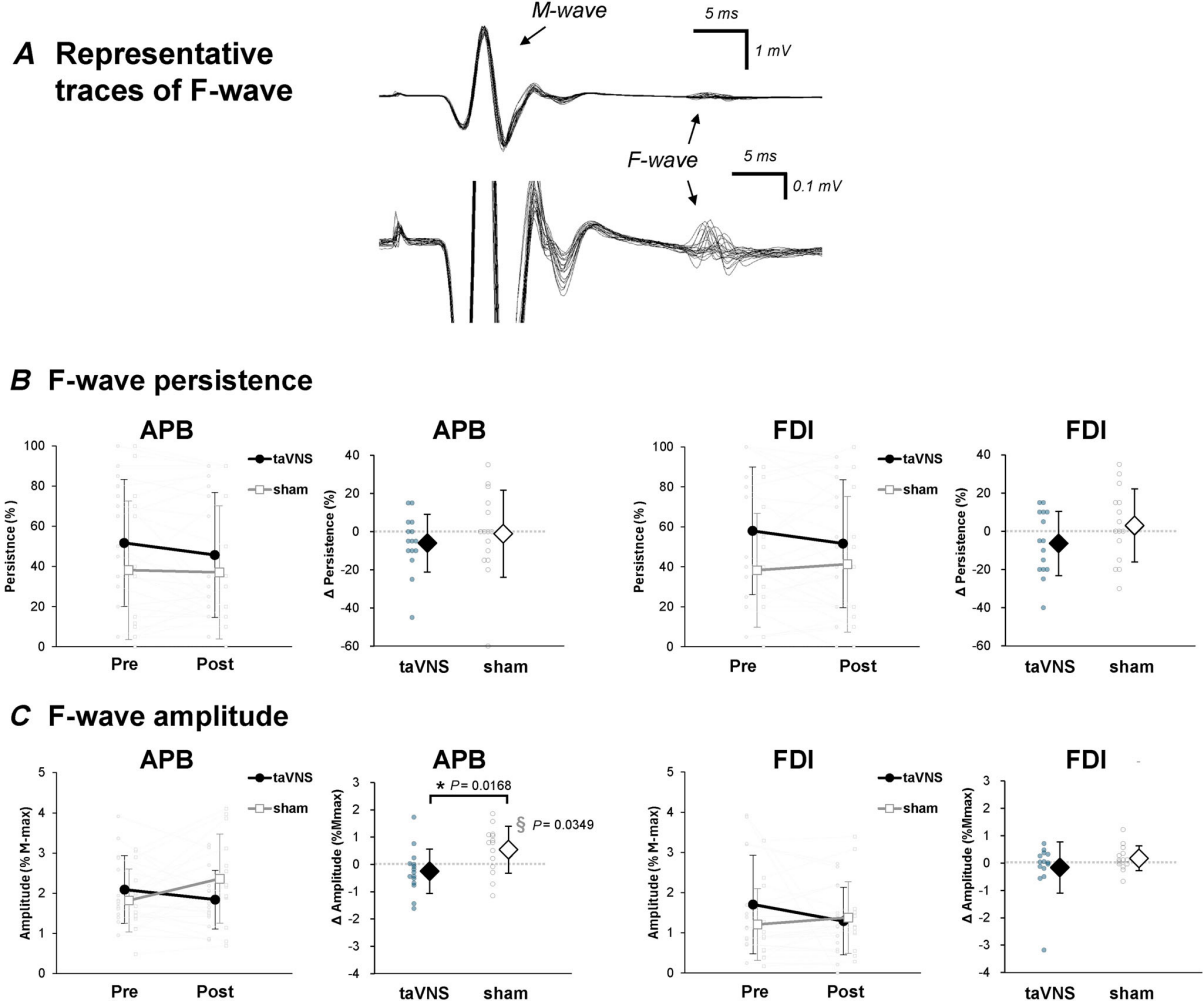
F‐wave measurement and results *A*, representative M‐wave and F‐wave traces from APB (20 trials, Pre‐test). *B*, group data for F‐wave persistence. *C*, group data for F‐wave amplitude (F/M ratio). In the Pre–Post panels, small symbols represent individual participants (circles: taVNS; squares: sham), with lines connecting the Pre and Post values within each participant. Large symbols represent group means. Error bars indicate ±SD. *taVNS *versus* sham; §Δ *versus* zero.

MEP amplitude was quantified as the peak‐to‐peak value within a 20–50 ms window after TMS (van de Ruit et al., 2015). Motor map characteristics were evaluated in two ways: (1) map size, defined as the number of active points, which reflects the spatial extent of the representation (Maeo et al., 2021; Nakagawa et al., 2020), and (2) map volume, defined as the sum of the mean MEP amplitudes across all active points, providing information on the overall excitability of the cortical representation (Maeo et al., 2021; Rossini et al., 2015).

### F‐wave measurement

F‐waves are widely used as an index of spinal motoneuron pool excitability because they reflect antidromic activation and backfiring of α‐motoneurons without involving synaptic transmission through sensory afferents. F‐waves were elicited by electrical stimulation of the median nerve approximately 8 cm proximal to the left wrist, using 3.2‐cm circular electrodes (cathode distal, anode proximal) connected to a constant‐current stimulator (DS7A, Digitimer) (Fig. 2). The inter‐electrode distance was approximately 5 cm. Prior to electrode attachment, the optimal stimulation site was identified with a probe electrode as the location producing clear M‐waves in both APB and FDI muscles. Participants were seated comfortably with their left hand supinated on a desk.

For each test (Pre‐ and Post‐test), twenty stimuli (200 µs pulse width) were delivered at 0.5 Hz at 120% of the intensity required to evoke the maximum M‐wave amplitude (M‐max) in the APB. One participant in the taVNS group did not undergo F‐wave measurement because of pain intolerance.

For F‐wave analysis, an additional high‐pass filter at 25 Hz was applied offline to suppress the trailing edge of the preceding M‐wave (Kaneko et al., 2022). As F‐wave latency varies across and within participants, the analysis window for peak‐to‐peak amplitudes was adjusted individually for each trial by visual inspection. Spinal excitability was assessed using two parameters: (1) F‐wave persistence, defined as the percentage of detectable F‐waves out of 20 stimuli (detection threshold: 0.03 mV) (Wupuer et al., 2013), and (2) F‐wave amplitude, defined as the mean peak‐to‐peak amplitude of detectable F‐waves normalised to M‐max (F/M ratio). If no F‐wave was detected across the 20 trials, the amplitude data for that participant were excluded from statistical analysis.

### Statistical analysis of behaviour, MEP and F‐wave

Data normality was assessed using the Shapiro–Wilk test. When normality was confirmed, two‐way repeated measures analysis of variance (ANOVA) (group [taVNS, sham] × time [Pre, Post]) were performed. When a significant interaction was found, *post hoc* tests were performed: Student's unpaired *t* test for between‐group comparisons (taVNS *vs*. sham) and a paired *t* test for within‐group comparisons (Pre *vs*. Post). For these datasets with four comparisons, the significance level was adjusted to *P* < 0.0125 using Bonferroni correction. When normality was violated, non‐parametric tests were applied (Mann–Whitney *U*‐test for between‐group comparisons and Wilcoxon's signed‐rank test for within‐group comparisons).

Additionally, the magnitude of change (Δ) from Pre‐ to Post‐test was calculated for each parameter. Between‐group comparisons of Δ values were performed using an unpaired *t* test (for normally distributed data) or the Mann–Whitney *U*‐test (for non‐normal data). Within‐group Δ values were compared against zero using a one‐sample *t* test (parametric) or Wilcoxon's signed‐rank test (non‐parametric). For analyses of Δ values, significance was set at *P* < 0.05.

### Measurements and analysis of pupillary response

During the practice session (task and rest periods), pupil diameter was recorded at 120 Hz from the left eye using an eye tracker (Pupil Core, Pupil Labs, Germany) (Fig. 2). The tracker was mounted immediately before the first practice set and removed after the final set. All recordings were conducted in the same experimental room under stable artificial lighting conditions with no external light sources, ensuring consistent illumination across sessions and participants.

Pupil data were pre‐processed in MATLAB R2021a following the guidelines of Kret & Sjak‐Shie (2019). Only samples with a confidence value ≥0.7 were retained. Values outside the physiologically plausible range of 1.5–9 mm were discarded. Blink‐related artifacts were identified using the first derivative of the pupil signal (dilation speed). Samples exhibiting the median + 3 median absolute deviations (MAD) were classified as artifacts, and an additional 100 ms (three samples before and after) was removed to capture the full blink. Continuous gaps >75 ms were expanded by 50 ms on each side. Further noise artifacts were detected by comparing the raw signal with a moving‐average smoothed version; deviations >3 MAD were excluded. Missing data were linearly interpolated only for gaps ≤0.5 s. The resulting signal was resampled to 30 Hz and smoothed with a zero‐phase low‐pass Butterworth filter (4 Hz cutoff).

Data were segmented into task and rest periods. For each period, pupil diameter was baseline corrected to the mean of the 0.2 s preceding the task/rest switch (stimulation on/off in taVNS group) (Wienke et al., 2023) and expressed as percentage change. Values were averaged across the 30 task or rest periods for each participant.

Time series differences in pupil diameter were examined using statistical parametric mapping for one‐dimensional data (SPM1D, www.spm1d.org) implemented in MATLAB (Meissner et al., 2024). A one‐sample *t* test against baseline (0) was performed within each condition. A paired *t* test compared task *versus* rest within each group, and an unpaired *t* test compared the taVNS group and the sham group under the same condition. At each time point, *t*‐ and *P*‐values were computed, with significance set at α = 0.05 (two‐tailed). To ensure physiological relevance, only clusters >3 consecutive time points (>0.1 s at 30 Hz; Wierda et al., 2012) exceeding the critical threshold were considered significant. Analyses were restricted to 0.2–25.0 s of 30‐s trial, excluding the first 0.2 s (latency of pupil response) (Mathôt, 2018) and the final 5 s (anticipatory effects from focusing on the timer or task completion). In addition, the velocity of the change of pupil size was compared between the groups to assess potential differences in response dynamics. As a control analysis, non‐normalised (absolute) baseline pupil diameter for each phase (task and rest) was compared between the groups using an unpaired *t* test to confirm the absence of baseline differences. Two participants (taVNS group: *n* = 1, sham group: *n* = 1) were excluded owing to eye tracker malfunction.

No AI tool has been used in the preparation of this manuscript.

## Results

### Motor performance (Fig. 3)

Figure 3*A* shows representative displacement traces of the two balls along the *y*‐axis during clockwise (CW) rotation in the Pre‐ and Post‐tests. Compared with the Pre‐test, rotation velocity and stability increased in the Post‐test.

For the number of rotations, Shapiro–Wilk tests confirmed normality in all datasets. Two‐way ANOVAs revealed a significant main effect of time (CCW: *F*(1, 29) = 49.397, *P* < 0.001, $\eta_{p}^{2}$ = 0.630); CW: *F*(1, 29) = 127.18, *P* < 0.001, $\eta_{p}^{2}$ = 0.814), but no significant main effect of group (CCW: *F*(1, 29) = 0.084, *P* = 0.773, $\eta_{p}^{2}$ = .003; CW: *F*(1, 29) = 0.563, *P* = 0.459, $\eta_{p}^{2}$ = 0.019) or group × time interaction (CCW: *F*(1, 29) = 0.175, *P* = 0.365, $\eta_{p}^{2}$ = 0.006; CW: *F*(1, 29) = 0.070, *P* = 0.794, $\eta_{p}^{2}$ = 0.002) (Fig. 3*B*).

For the rotation instability, non‐normality was detected in several datasets (e.g. taVNS_Post in CCW, sham_Pre in CCW, taVNS_Pre in CW). Subsequent analyses showed that instability in CW rotations significantly decreased from Pre‐ to Post‐test in both the taVNS (*Z =* 3.516, *P* < 0.001, *r = *0.622) and sham groups (*t*(14) = 5.698, *P* < 0.001, *d* = 1.866). For CW rotation, there was no significant difference between taVNS and sham groups at either Pre‐test (*Z =* 0.514, *P* = 0.626, *r* = 0.0923) or Post‐test (*t*(29) = 2.11, *P* = 0.0433, *d* = 0.760). For CCW rotations, instability significantly decreased from Pre‐ to Post‐test in the taVNS group (*Z =* 2.64, *P* = 0.00818, *r* = 0.467) but not in the sham group (*Z =* 1.704, *P* = 0.088, *r* = 0.310). There was no significant difference in CCW instability between taVNS and sham groups at either Pre‐test (*Z =* 1.146, *P* = 0.264, *r* = 0.207) or Post‐test (*Z =* 0.100, *P* = 0.922, *r* = 0.018) (Fig. 3*C*).

### Motor representation by TMS mapping

The number of stimulated grid points (mean ± SD) was 42.9 ± 11.3 at Pre and 47.3 ± 14.7 at Post in the taVNS group, and 47.1 ± 10.9 at Pre and 42.1 ± 11.9 at Post in the sham group.

### Map size (Fig. 4*C*)

For the APB muscle, the Shapiro–Wilk test indicated non‐normality in the taVNS_Pre and taVNS_Post datasets. No significant differences were detected in any of the pairwise comparisons: taVNS_Pre *versus* taVNS_post (*Z =* 1.052, *P* = 0.293, *r* = 0.186), sham_Pre *versus* sham_Post (*t*(14) = 1.504, *P* = 0.155, *d* = 0.348), taVNS_Pre *versus* sham_Pre (*Z =* 0.813, *P* = 0.423, *r* = 0.146) and taVNS_Post *versus* sham_Post (*Z =* 0.456, *P* = 0.654, *r* = 0.082). For the Δ map size, normality was confirmed in both groups, and unpaired *t* tests revealed that Δ map size was significantly greater in the taVNS group than in the sham group (*t*(29) = 2.081, *P* = 0.0464, *d* = 0.748). The Δ map sizes did not significantly differ from zero in either the taVNS (*t*(15) = 1.437, *P* = 0.171, *d* = 0.508) or sham group (*t*(14) = 1.504, *P* = 0.155, *d* = 0.549).

For the FDI muscle, non‐normality was detected in the taVNS_Post datasets, and no significant differences were found in any of the pairwise comparisons: taVNS_Pre *versus* taVNS_post (*Z =* 0.683, *P* = 0.495, *r* = 0.121), sham_Pre *versus* sham_Post (*t*(14) = 1.400, *P* = 0.184, *d* = 0.227), taVNS_Pre *versus* sham_Pre (*t*(29) = 0.169, *P* = 0.867, *d* = 0.061) and taVNS_Post *versus* sham_Post (*Z =* 0.436, *P* = 0.682, *r* = 0.0783). For the Δ map size, normality was confirmed in both groups, but no significant group differences were observed (*t*(29) = 1.657, *P* = 0.108, *d* = 0.600). The Δ map sizes did not significantly differ from zero in either the taVNS (*t*(15) = 0.988, *P* = 0.339, *d* = 0.349) or the sham group (*t*(14) = 1.400, *P* = 0.184, *d* = 0.510).

### Map volume (Fig. 4*D*)

For the APB muscle, non‐normality was observed in the taVNS_Pre and taVNS_Post datasets, and no significant differences were found in any of the pairwise comparisons: taVNS_Pre *versus* taVNS_post (*Z =* 1.500, *P* = 0.134, *r* = 0.269), sham_Pre *versus* sham_Post (*Z =* 1.477, *P* = 0.140, *r* = 0.270), taVNS_Pre *versus* sham_Pre (*Z =* 1.324, *P* = 0.188, *r* = 0.237) and taVNS_Post *versus* sham_Post (*Z =* 0.178, *P* = 0.861, *r* = 0.0320). For the Δ map volume, normality was not confirmed in the taVNS group. A Mann–Whitney *U*‐test showed that Δ map volume was significantly greater in the taVNS group than in the sham group (*Z =* 2.253, *P* = 0.0240, *r* = 0.405). The Δ map volumes did not significantly differ from zero in either the taVNS (*Z =* 1.474, *P* = 0.140, *r* = 0.261) or the sham group (*t*(14) = 1.470, *P* = 0.164, *d* = 0.537).

For the FDI muscle, non‐normality was detected in the taVNS_Pre and taVNS_Post datasets, with no significant pairwise differences: taVNS_Pre *versus* taVNS_Post (*Z =* 1.241, *P* = 0.215, *r* = 0.219), sham_Pre *versus* sham_Post (*t*(14) = 2.420, *P* = 0.0297, *d* = 0.442), taVNS_Pre *versus* sham_Pre (*Z =* 0.929, *P* = 0.358, *r* = 0.167) and taVNS_Post *versus* sham_Post (*Z =* 0.494, *P* = 0.626, *r* = 0.088). For the Δ map volume, normality was not confirmed in the taVNS group. A Mann–Whitney *U*‐test indicated that Δ map volume was significantly greater in the taVNS group than in the sham group (*Z =* 2.471, *P* = 0.0120, *r = *0.444). In addition, the Wilcoxon signed‐rank test showed that Δ map volume in the sham group was significantly less than zero (*Z =* 2.385, *P* = 0.0171, *r = *0.436). The Δ map volumes in the taVNS did not significantly differ from zero (*Z =* 1.242, *P* = 0.214, *r = *0.219).

### Spinal excitability by F‐wave measurements

#### F‐wave persistence (Fig. 5*B*)

For the APB muscle, the Shapiro–Wilk test indicated non‐normality in several datasets (taVNS_Post, sham_Pre and sham_Post). No significant differences were found in pairwise comparisons: taVNS_Pre *versus* taVNS_Post (*Z =* 1.375, *P* = 0.169, *r* = 0.243), sham_Pre *versus* sham_Post (*Z =* 1.341, *P* = 0.180, *r* = 0.245), taVNS_Pre *versus* sham_Pre (*Z =* 1.185, *P* = 0.250, *r* = 0.213) and taVNS_Post *versus* sham_Post (*Z =* 0.939, *P* = 0.367, *r* = 0.0169). For the Δ persistence, normality was confirmed in both groups, and unpaired *t* tests showed no significant group differences (*t*(27) = 0.694, *P* = 0.494, *d* = 0.260). The Δ persistence did not significantly differ from zero in either the taVNS (*t*(14) = 1.535, *P* = 0.147, *d* = 0.560) or the sham group (*t*(13) = 0.187, *P* = 0.855, *d* = 0.068).

For the FDI muscle, non‐normality was detected in the sham_Post dataset, and no significant differences were observed in any of the pairwise comparisons (*t*(14) = 1.535, *P* = 0.147, *d* = 0.192), sham_Pre *versus* sham_Post (*Z =* 0.551, *P* = 0.582, *r* = 0.104), taVNS_Pre *versus* sham_Pre (*t*(27) = 1.783, *P* = 0.0859, *d* = 0.650) and taVNS_Post *versus* sham_Post (*Z =* 0.897, *P* = 0.389, *r* = 0.0167). Similarly, Δ persistence values showed no significant group differences (*t*(27) = 1.418, *P* = 0.168, *d* = 0.520). The Δ persistence did not significantly differ from zero in either the taVNS (*t*(14) = 1.456, *P* = 0.167, *d* = 0.532) or the sham group (*t*(13) = 0.608, *P* = 0.554, *d* = 0.221).

#### F‐wave amplitude (Fig. 5*C*)

For the APB muscle, all datasets met normality assumptions. A two‐way ANOVA revealed a significant group × time interaction (*F*(1, 27) = 6.49, *P* = 0.017, $\eta_{p}^{2}$ = 0.194), with no main effects of group (*F*(1, 27) = 0.190, *P* = 0.666, $\eta_{p}^{2}$ = 0.007) or time (*F*(1, 27) = 0.872, *P* = 0.358, $\eta_{p}^{2}$ = 0.031). However, follow‐up paired *t* tests did not identify significant differences among the four individual conditions: taVNS_Pre *versus* taVNS_Post (*t*(14) = 1.194, *P* = 0.252, *d* = 0.317), sham_Pre *versus* sham_Post (*t*(13) = 2.355, *P* = 0.0349, *d* = 0.562), taVNS_Pre *versus* sham_Pre (*t*(27) = 0.891, *P* = 0.381, *d* = 0.330) and taVNS_Post *versus* sham_Post (*t*(27) = 1.502, *P* = 0.145, *d* = 0.560). For Δ amplitude, unpaired *t* tests showed that values were significantly greater in the sham group than in the taVNS group (*t*(27) = 2.548, *P* = 0.0168, *d* = 0.950). Additionally, the sham group exhibited a significant increase in Δ amplitude compared with zero (*t*(13) = 2.355, *P* = 0.0349, *d* = 0.890). The Δ amplitude in the taVNS group did not differ from zero (*t*(14) = 1.194, *P* = 0.252, *d* = 0.436).

For the FDI muscle, no significant differences were detected in pairwise comparisons: taVNS_Pre *versus* taVNS_Post (*Z =* 0.284, *P* = 0.776, *r* = 0.0519), sham_Pre *versus* sham_Post (*Z =* 1.412, *P* = 0.158, *r* = 0.267), taVNS_Pre *versus* sham_Pre (*Z =* 1.091, *P* = 0.290, *r* = 0.203) and taVNS_Post *versus* sham_Post (*Z =* 0.218, *P* = 0.847, *r* = 0.0405). For the Δ amplitude values, there was no significant group‐difference (*Z =* 0.850, *P* = 0.401, *r* = 0.158). Furthermore, the Δ amplitude did not significantly differ from zero in either the taVNS (*Z =* 0.126, *P* = 0.900, *r* = 0.023) or the sham group (*t*(13) = 1.42, *P* = 0.179, *d* = 0.537).

#### Pupil dilation (Fig. 6*A*)

**Figure 6:**
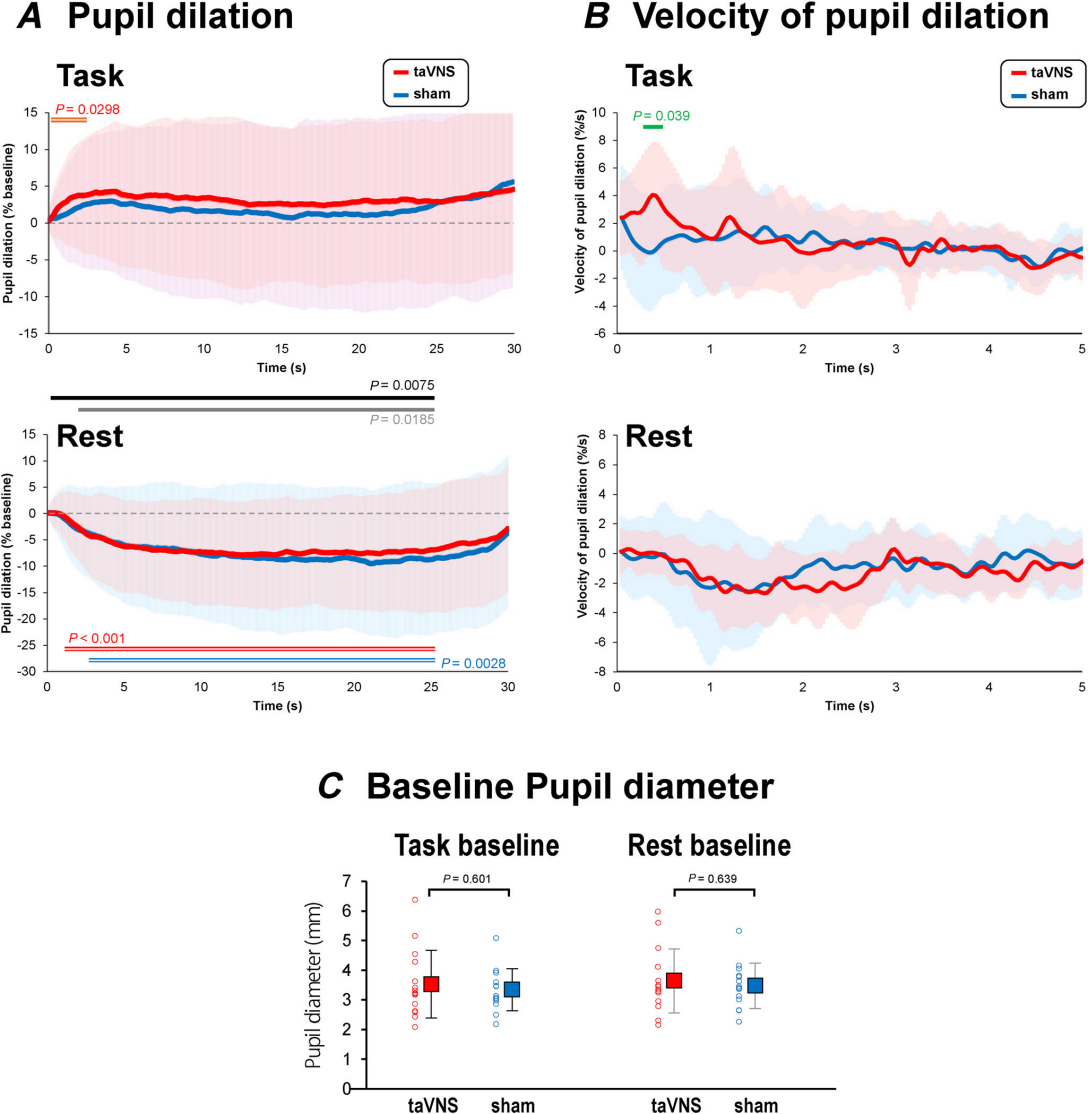
Pupil dilation during task and rest *A*, grand‐average pupil diameter (percentage change from baseline) during task (top) and rest (bottom). Solid lines: taVNS (red), sham (blue); shaded areas indicate ±SD. Red double lines indicate time ranges significantly different from baseline in the taVNS group; blue double lines indicate those in the sham group. Black and grey horizontal bars denote periods where task–rest differences were significant within the taVNS and sham groups, respectively. No significant differences were found between groups. *B*, grand‐average of velocity of pupil dilation in the early phase (0–5s). Green bar indicates the period with a significant difference between groups during the task. No significant group differences during rest. *C*, comparisons of absolute baseline pupil diameter (mm) between groups during task and rest baseline periods. In either task or rest phase baselines, they did not significantly differ between groups. Symbols of the large squares and small circles represent the mean value and individual data, respectively. Error bars indicate ±SD.

Figure 6*A* shows the normalised pupil diameter from the onset of task/rest periods (0 s) to 30 s. During the task period, the taVNS group exhibited a significant pupil dilation relative to baseline between 0.2 and 2.3 s (cluster *P* = 0.0298), whereas no significant change was observed in the sham group. During the rest period, both groups showed significant pupil constriction relative to baseline: 1.2–25.0 s for the taVNS (cluster *P* < 0.001) and 2.8–25.0 s for the sham group (cluster *P* = 0.0028). Direct comparisons between task and rest revealed significant differences in the VNS group across 0.2–25.0 s (cluster *P* = 0.0075), and in the sham group across 2.1–25.0 s (cluster *P* = 0.0185).

#### Velocity of pupil dilation (Fig. 6*B*)

Figure 6*B* illustrates the velocity of pupil size changes during the first 5 s after onset. During the task period, the the taVNS group showed significantly faster pupil dilation than did the sham group at 0.3–0.47 s after onset (cluster *P* = 0.039).

#### Absolute baseline pupil diameter (Fig. 6*C*)

Figure 6*C* shows baseline pupil diameters for each phase and group. Absolute baseline pupil diameter did not differ between groups during either the task baseline period (taVNS *versus* sham, *t*(27) = 0.529, *P* = 0.601, *d* = 0.200) or the rest baseline period (taVNS *versus*. sham, *t*(27) = 0.475, *P* = 0.639, *d* = 0.180), indicating no baseline differences between conditions that would confound the interpretation of pupil responses.

#### Validation of the three‐stimulus TMS mapping protocol

To validate the use of three stimuli per grid point in the main experiment, we conducted an additional validation experiment comparing corticomotor maps obtained using three *versus* five stimuli per grid point.

Ten healthy participants (27 ± 4 years, mean ± SD; four females; two left‐handed) underwent additional mapping using both three‐ and five‐stimulus protocols in a counterbalanced order within the same day. Map size and map volume were calculated using the same criteria as in the main experiment. The association between protocols was assessed using Pearson correlation coefficients. Agreement was evaluated using Bland–Altman analysis, including estimation of the mean difference (bias) and limits of agreement (mean ± 1.96 SD). Scatter plots with identity lines (*y* = *x*) were used to visualise the relationship between the two measurements.

**Figure 7:**
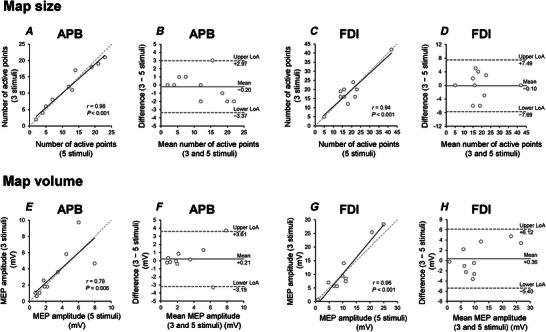
Validation of TMS mapping outcomes obtained with three *versus* five stimuli per grid point in APB and FDI muscles Scatter plots and corresponding Bland–Altman plots comparing TMS mapping outcomes derived from 3 and 5 stimuli per grid point (*n* = 10). Map size (number of active grid points) is shown for APB in panels *A* and *B*, and for FDI in panels *C* and *D*. Map volume (sum of MEP amplitudes) is shown for APB in panels *E* and *F*, and for FDI in panels *G* and *H*. In scatter plots *A*, *C*, *E* and *G*, the continuous line represents the linear regression and the dashed line indicates the line of identity (*y* = *x*). Pearson correlation coefficients (*r*) and corresponding *P*‐values are displayed in each panel. In Bland–Altman plots *B*, *D*, *F* and *H*, the difference between protocols (3−5 stimuli) is plotted against the mean of the two measures. The solid horizontal line represents the mean difference (bias), and the dashed lines indicate the upper and lower limits of agreement (mean difference ± 1.96 SD). Across both muscles (APB and FDI) and outcome measures, strong correlations and minimal systematic bias were observed, supporting the validity of the 3‐stimulus protocol under the present mapping conditions.

Strong correlations were observed between the three‐ and five‐stimulus protocols (APB map size: *r* = 0.98, *P* < 0.001 (Fig. 7*A*); FDI map size: *r* = 0.94, *P* < 0.001 (Fig. 7*C*); APB map volume: *r* = 0.79, *P* = 0.006 (Fig. 7*E*); FDI map volume: *r* = 0.96, *P* < 0.001 (Fig. 7*G*)). Figure 7*B*, *D*, *F* and *H* show the Bland–Altman plots. They showed minimal bias with mean differences close to zero. The limits of agreement were small relative to the observed range, indicating good practical agreement between protocols.

These findings indicate that corticomotor map size and volume obtained using three stimuli per grid point provide comparable estimates to those obtained with the conventional five‐stimulus protocol under the present mapping conditions.

## Discussion

This study aimed to investigate whether short‐term taVNS paired with periodic motor skill practice enhances motor learning and promotes plasticity in motor‐related neural circuits. Our results indicate that taVNS during two‐ball rotation practice facilitated the expansion of M1 representation of the finger muscles, whereas motor learning itself was not enhanced. Moreover, F‐wave amplitude increased in the sham group but not in the taVNS group, consistent with increased spinal motoneuron excitability following motor practice in the absence of stimulation. Finally, the taVNS group exhibited stronger pupil dilation during practice, consistent with greater recruitment of the LC‐NA system.

### Effects of taVNS on motor learning

Both groups significantly improved in two‐ball rotation performance, but taVNS did not provide additional behavioural benefit. Thus, our hypothesis was not supported at the behavioural level. One possible explanation is the brevity of the intervention: only 15 min of practice with stimulation. In contrast, clinical studies that demonstrated functional gains in stroke patients typically involved intervention periods ranging from days to weeks (Gerges et al., 2024). Our findings suggest that the present protocol was sufficient to induce neural plasticity but insufficient to drive behavioural gains. Consistent with our findings, an animal study reported that VNS paired with motor training induced expansion of M1 maps without parallel performance gains (Hulsey et al., 2016), implying that cortical reorganization and behavioural improvements can occur on different timescales. Longer interventions may reveal behavioural benefits, as many studies demonstrating functional improvements with VNS have employed training protocols lasting from days to weeks (Gerges et al., 2024).

A second possibility is that the continuous ball‐rotation task did not provide explicit signals of success, unlike paradigms where VNS was paired with discrete, rewarded movements (Bowles et al., 2022). Although each rotation could technically be considered a success, participants may not have perceived the trial‐by‐trial outcomes, which could reduce the engagement of reward‐related neuromodulatory circuits. Motivation and task salience, typically high in rehabilitation or animal models, may have been relatively low here, attenuating the neuromodulatory influence of taVNS.

Alternatively, the effects of taVNS on motor learning may depend on the type of motor task. In the present study, a skilled continuous task (two‐ball rotation) was used, but a recent study reported that taVNS facilitated the motor adaptation in a force field learning paradigm (Obata & Sekiguchi, 2025). Notably, force‐field adaptation depends heavily on cerebellar function, and taVNS has been shown to activate the cerebellum in humans (Badran et al., 2018). This suggests a plausible mechanism by which taVNS could enhance cerebellum‐dependent learning processes. Another key distinction lies in the locus of plasticity associated with different task types. Motor adaptation has been thought to involve primarily cerebellar and cortical mechanisms, although the relative contribution of spinal circuits may vary depending on the specific task and training conditions (Ahmadi‐Pajouh et al., 2012; Ito et al., 2021). Indeed, our results suggest that the two‐ball rotation task engaged spinal plasticity, as indicated by increased F‐wave amplitude in the sham group following training. Although brainstem nuclei influenced by vagal afferents possess descending projections to the spinal cord (Brownstone & Chopek, 2018; Proudfit & Clark, 1991), taVNS primarily engages supraspinal neuromodulatory systems. Therefore, the expression of plasticity in the present task may have been biased toward supraspinal circuits, which could partly explain the limited behavioural benefit observed in this task. Taken together, these findings imply that taVNS may be more effective for tasks that rely on cerebellar or cortical plasticity rather than spinal mechanisms. Future studies should systematically compare different types of motor learning to determine which are most susceptible to taVNS‐induced behavioural enhancement.

Notably, only the taVNS group exhibited improved stability in the non‐practiced CCW condition. Given the greater M1 expansion observed in this group, this effect may reflect generalised learning through M1 reorganization, consistent with reports that non‐invasive M1 stimulation promotes intracortical disinhibition and motor generalization (Dumel et al., 2018).

### Effects of taVNS on M1 plasticity

Our results indicated greater expansion of M1 representations in the taVNS group than in the sham group, as reflected by larger changes in map size and map volume, supporting our cortical‐level hypothesis. This finding aligns with prior animal studies (Hulsey et al., 2016; Porter et al., 2012) and provides the first evidence in humans that taVNS can enhance motor map plasticity. Notably, the intervention duration was much shorter than in previous animal or clinical studies (ranging from days to weeks) (Gerges et al., 2024; Hulsey et al., 2016; Porter et al., 2012), highlighting the potency of taVNS to induce rapid cortical reorganization, which is highly relevant for clinical translation. In the sham group, most M1 parameters did not change significantly, except for a reduction in FDI map volume. This may reflect neural efficiency, a form of cortical plasticity where reduced activation supports improved skill execution (Callan & Naito, 2014). Therefore, taVNS may shift plasticity toward representational enlargement. It remains unclear whether this expansion is muscle‐specific or broader, as only APB and FDI were examined.

One methodological consideration relates to the number of TMS stimuli delivered at each grid point during motor mapping. In the present study, three stimuli were used per site to minimise the duration of post‐intervention assessment because the temporal stability of taVNS‐induced plasticity following short‐term training was unknown. Although previous studies have recommended a minimum of five stimuli to maximise mapping reliability (Cavaleri et al., 2017), an additional validation experiment demonstrated strong agreement between three‐ and five‐stimulus protocols under the present mapping conditions (Fig. 7). Importantly, the primary mapping outcomes analysed in this study (map size and map volume) represent aggregated measures derived across multiple grid points rather than single‐trial MEP amplitudes. Nevertheless, it remains possible that a larger number of stimuli could further improve measurement stability.

### Effects of taVNS on spinal motoneuron excitability

To explore plasticity beyond the brain, we measured F‐waves as an index of spinal motoneuron excitability. This approach enabled us to examine plastic changes at both cortical and spinal levels within the same experimental framework and provides insight into the hierarchical expression of plasticity in the motor system. In the sham group, F‐wave amplitude increased in APB, indicating use‐dependent plasticity at the spinal level. Such spinal adaptations have been reported after training of cyclic or postural skills (Harel et al., 2015; Mazzocchio et al., 2006), which can be performed unconsciously and where spinal contributions are likely substantial compared with the case of discrete movement. In contrast, such spinal‐level changes were not observed when practice was paired with taVNS. These findings indicate that the locus at which practice‐related plasticity was expressed differed between conditions.

Vagal afferents project to the nucleus of the solitary tract (NTS), which innervates multiple brainstem nuclei including the locus coeruleus (LC) and basal forebrain (Rodenkirch et al., 2022), thereby modulating cortical plasticity via ascending neuromodulatory systems. At the same time, LC neurons send noradrenergic descending projections to the spinal cord, including the ventral horn and intermediate zone, suggesting a potential route by which taVNS‐related neuromodulatory signals could influence spinal circuitry (Proudfit & Clark, 1991). In addition, reticulospinal neurons arising from the pontomedullary reticular formation project to spinal interneurons and motoneurons involved in posture and voluntary movement, and are known to contribute to the coordination of muscle activation patterns and to the tuning of descending motor commands during voluntary movement (Brownstone & Chopek, 2018). These pathways may influence the overall excitability state of the spinal motor system during voluntary tasks, thereby indirectly shaping the expression of spinal plasticity. Thus, taVNS may influence the level at which plasticity is expressed within the motor system, spanning both cortical and spinal circuits.

However, under the present short‐term intervention we observed cortical reorganization without detectable changes in F‐wave indices in the taVNS group. Taken together, these results suggest that neuromodulatory context may influence the level at which expression of plasticity is expressed within the motor system. That is, motor practice alone in this study may lead to measurable adaptation at the level of the motoneuron pool, whereas pairing practice with taVNS may have been associated with a relative shift in the measurable expression of plasticity toward supraspinal circuits. This interpretation regarding the shift in the measurable expression of plasticity should be considered in the context of the brief intervention used in the present study and may not extend to other taVNS paradigms. We do not exclude the possibility that additional spinal mechanisms were engaged but not captured by F‐wave measurements. Furthermore, it should be noted that both TMS mapping and F‐wave measurements provide indirect indices of neural plasticity and cannot definitively localise the underlying synaptic mechanisms. Therefore, conclusions regarding the hierarchical locus of plasticity should be interpreted with appropriate caution.

If taVNS preferentially facilitates cortical plasticity, its therapeutic potential may depend on the locus of neural dysfunction. This may partly explain why several clinical studies reporting beneficial effects of taVNS have been reported in conditions involving supraspinal dysfunction (e.g. stroke) (Ahmed et al., 2023; Gerges et al., 2024), although further work is needed to determine whether similar benefits extend to disorders primarily involving spinal pathology.

### Effects of taVNS on pupil size

To our knowledge, this study is the first to examine pupil dynamics during motor practice with taVNS. Both groups showed task‐related pupil dilation relative to rest; however, only the taVNS group exhibited a rapid and significant dilation immediately after task onset, accompanied by faster dilation velocity, than did the sham group. Pupil dilation also began several seconds before the switch from rest to task, likely reflecting predictive preparation, as the timing of stimulation onset was displayed on the monitor. Consequently, pupil size during the task period differed little from the immediate pre‐task baseline, except for a brief interval of significant dilation observed only in the taVNS group. Pupil diameter and its rate of change are reliable markers of LC‐NA system activity (Reimer et al., 2016), and thus these results indicate stronger noradrenergic engagement under taVNS. Because pupil size is also a proxy for cognitive states such as arousal, which are modulated by the LC‐NA system during exercise and motor learning (Kuwamizu et al., 2022; Yokoi & Weiler, 2022), it is possible that taVNS participants were in a state of greater attentional focus on the task, which may in turn have contributed to the observed M1 plasticity.

### Possible mechanisms of M1 plastic changes by the combination of motor practice and taVNS

Previous studies demonstrated that the two‐ball rotation task engages M1, premotor, supplementary motor and cerebellar regions (Matsumura et al., 2004; Park et al., 2008). In parallel, taVNS activates the LC and cerebellum even at rest (Badran et al., 2018; Frangos et al., 2015) and modulates cerebello–thalamo–M1 pathways (van Midden et al., 2024). Thus, combined activation of cerebellar circuits during practice and taVNS may have facilitated M1 reorganization via the LC–cerebellum–thalamus–M1 pathway. Although short‐term practice was insufficient to improve behaviour, longer interventions may strengthen cerebellar internal models and promote learning.

**Figure 8:**
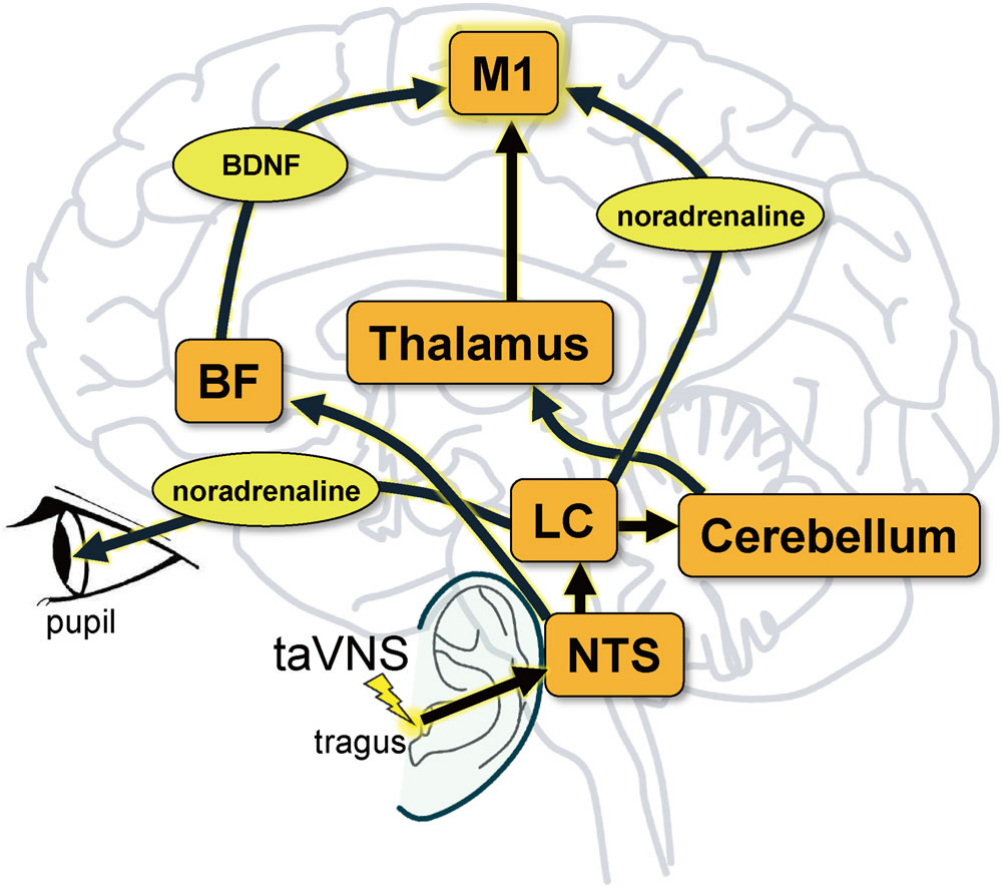
Putative neural pathways underlying M1 plasticity induced by the combination of motor practice and taVNS Transcutaneous auricular vagus nerve stimulation (taVNS) activates brainstem regions including the nucleus of the solitary tract (NTS), which projects to the locus coeruleus (LC) and basal forebrain (BF), as well as to the cerebellum. These pathways are proposed to influence primary motor cortex (M1) plasticity via neuromodulatory systems (e.g. noradrenergic and cholinergic projections) and cerebello–thalamo–cortical circuits. Arrows indicate putative pathways through which taVNS may influence cortical plasticity, including both direct projections and neuromodulatory influences.

At the molecular level, VNS has been shown to enhance brain‐derived neurotrophic factor (BDNF) expression (Hays et al., 2013). Recent animal work indicates that taVNS similarly increases cortical BDNF via cholinergic receptors (Li et al., 2020), while human studies suggest cholinergic recruitment by taVNS (Horinouchi et al., 2024). The cholinergic system has been identified as a key mediator of both motor learning (Bowles et al., 2022) and plastic expansion of M1 representation (Hulsey et al., 2016). In addition, noradrenergic engagement, as supported by our pupil findings, is likely critical (Lloyd et al., 2023; Pervaz et al., 2025; Sharon et al., 2021). Both LC‐NA and basal forebrain cholinergic systems receive projections from the NTS, the first relay of vagal afferents (Rodenkirch et al., 2022). Thus, taVNS probably co‐activates these systems, leading to increased release of neuromodulators and BDNF, thereby facilitating M1 plasticity. These mechanisms, illustrated schematically in Fig. 8, may collectively explain how taVNS paired with motor practice enhances neural plasticity in M1.

In summary, taVNS paired with dexterous motor practice did not enhance motor learning in the short term but selectively promoted reorganization of the M1 map without detectable spinal plastic change. Pupil dynamics indicated heightened noradrenergic engagement, suggesting differential recruitment of neuromodulatory circuits between groups. Although it did not significantly boost behavioural improvement in the short term, taVNS may provide a neural substrate for long‐term functional recovery when combined with extended training. These findings highlight taVNS as a selective modulator of cortical plasticity in humans and support its potential as a targeted adjunct to neurorehabilitation.

## Additional information

## Competing interests

The authors declare no competing financial interests.

## Author contributions

K.N. and R.O. conceived and designed research, interpreted results and edited the manuscript. K.N. performed experiment, analysed data and drafted the manuscript. Both authors have read and approved the final version of this manuscript and agree to be accountable for all aspects of the work in ensuring that questions related to the accuracy or integrity of any part of the work are appropriately investigated and resolved. All persons designated as authors qualify for authorship, and all those who qualify for authorship are listed.

## Funding

This study was supported by Grant‐in‐Aid for Scientific Research (B) (JSPS KAKENHI Grant Number 22H03498) to K.N., Grant‐in‐Aid for Scientific Research (A) (JSPS KAKENHI Grant Number 21H04425) to R.O., and Grant‐in‐Aid for Scientific Research (A) (JSPS KAKENHI Grant Number 23H00459) to R.O.

## Supporting information


Peer Review History

## Data Availability

Data are available from the corresponding author upon reasonable request.
