# Supplementary material for: Transcutaneous auricular vagus nerve stimulation during short‐term motor practice drives cortical plasticity without behavioural improvement
Source: J Physiol. 2026 Jun 19;604(14):6128–46. doi: 10.1113/JP290398 (PMC13370720; doi:10.1113/JP290398)
Supplement: Supplementary file 1 — Peer Review History [file TJP-604-6128-s001.pdf]

The Journal of Physiology

Transcutaneous auricular vagus nerve stimulation during short-term motor practice drives cortical plasticity without behavioural improvement

Kento Nakagawa and Rieko Osu
DOI: 10.1113/JP290398

Corresponding author(s): Kento Nakagawa (nakagawa@jobu.ac.jp)

The referees have opted to remain anonymous.

Review Timeline:

Submission Date:	25-Oct-2025
Editorial Decision:	24-Nov-2025
Revision Received:	26-Mar-2026
Editorial Decision:	14-Apr-2026
Revision Received:	30-Apr-2026
Editorial Decision:	21-May-2026
Revision Received:	22-May-2026
Accepted:	03-Jun-2026

Senior Editor: Richard Carson

Reviewing Editor: Jacob Thorstensen

Transaction Report:

(Note: With the exception of the correction of typographical or spelling errors that could be a source of ambiguity, letters and reports are not edited. Depending on transfer agreements, referee reports obtained elsewhere may or may not be included in this compilation. Referee reports are anonymous unless the Referee chooses to sign their reports.)

Re: JP-RP-2025-290398 "**Transcutaneous auricular vagus nerve stimulation during short-term motor practice drives cortical plasticity without behavioral improvement** " by Kento Nakagawa and Rieko Osu

Dear Dr Nakagawa,

Thank you for submitting your manuscript to The Journal of Physiology. It has been assessed by a Reviewing Editor and by 2 expert referees and we are pleased to tell you that it is potentially acceptable for publication following satisfactory major revision.

Please advise your co-authors of this decision as soon as possible.

The referee reports are copied at the end of this email.

Please address all the points raised and incorporate all requested revisions or explain in your Response to Referees why a change has not been made. We hope you will find the comments helpful and that you will be able to return your revised manuscript within 2 months. If your article is NOT for a Special Issue, you may have 9 months to revise. If you require an extension, please contact journal staff: jp@physoc.org. Please note that this letter does not constitute a guarantee for acceptance of your revised manuscript.

Your revised manuscript should be submitted online using the link in your Author Tasks: Link Not Available. This link is accessible via your account as Corresponding Author; it is not available to your co-authors. If this presents a problem, please contact journal staff (jp@physoc.org). Image files from the previous version are retained on the system. Please ensure you replace or remove any files that are being revised.

If you do not wish to submit a revised version of your manuscript, you must inform our journal staff (jp@physoc.org) or reply to this email to request withdrawal. Please note that a manuscript must be formally withdrawn from the peer review process at one journal before it may be submitted to another journal.

TRANSPARENT PEER REVIEW POLICY: To improve the transparency of its peer review process, The Journal of Physiology publishes online as supporting information the peer review history of all articles accepted for publication. Readers will have access to decision letters, including Editors' comments and referee reports, for each version of the manuscript, as well as any author responses to peer review comments. Referees can decide whether or not they wish to be named on the peer review history document.

ABSTRACT FIGURES: Authors are expected to use The Journal's premium BioRender account to create/redraw their Abstract Figures. Information on how to access this account is here: https://physoc.onlinelibrary.wiley.com/journal/14697793/biorender-access.

This will enable Authors to create and download high-resolution figures. If authors have used the free BioRender service, they can use the instructions provided in the link above to download a high-resolution version suitable for publication.

The link provided should only be used for the purposes of this submission. Authors will be charged for figures created on this account if they are not related to this manuscript submission.

LANGUAGE EDITING AND SUPPORT FOR PUBLICATION: If you would like help with English language editing, or other article preparation support, Wiley Editing Services offers expert help, including English Language Editing, as well as translation, manuscript formatting, and figure formatting at www.wileyauthors.com/eeo/preparation. You can also find resources for Preparing Your Article for general guidance about writing and preparing your manuscript at www.wileyauthors.com/eeo/prepresources.

RIGOUR AND REPRODUCIBILITY: We recommend authors consult The Journal's Rigour and Reproducibility requirements to ensure that their manuscript is suitable for publication: https://physoc.onlinelibrary.wiley.com/pb-assets/hub-assets/physoc/documents/TJP-Rigour-and-Reproducibility-Requirements.pdf

REVISION CHECKLIST:

Check that your Methods section conforms to journal policy: https://jp.msubmit.net/cgi-bin/main.plex?form_type=display_requirements#methods.

Check that data presented conforms to the statistics policy: https://jp.msubmit.net/cgi-bin/main.plex?form_type=display_requirements#statistics.

Upload a full Response to Referees file. To create your 'Response to Referees': copy all the reports, including any comments from the Senior and Reviewing Editors, into a Microsoft Word, or similar, file and respond to each point, using font or background colour to distinguish comments and responses and upload as the required file type.

Please upload two versions of your manuscript text: one with all relevant changes highlighted and one clean version with no changes tracked. The manuscript file should include all tables and figure legends, but each figure/graph should be uploaded as separate, high-resolution files.

You may also upload:

- 'Potential Cover Art' for consideration as the issue's cover image
- Appropriate Supporting Information (Video, audio or data set: see https://jp.msubmit.net/cgi-bin/main.plex?form_type=display_requirements#supp).

We look forward to receiving your revised submission.

If you have any queries, please reply to this email and we will be pleased to advise.

Yours sincerely,

Richard Carson
Senior Editor
The Journal of Physiology

REQUIRED ITEMS

1) - Author photo and profile. First or joint first authors are asked to provide a short biography (no more than 100 words for one author or 150 words in total for joint first authors) and a portrait photograph. These should be uploaded and clearly labelled together in a Word document with the revised version of the manuscript. See Information for Authors for further details.

2) - The contact information for the person responsible for 'Research Governance' at your institution needs to be provided. This includes their name and an institutional email address. Please ensure the contact is not an author on this paper and provide an alternate contact if necessary, or confirm in the submission form that the author whose email was provided has sole responsibility for research governance. This is the person who is responsible for regulations, principles and standards of good practice in research carried out at the institution, for instance the ethical treatment of animals, the keeping of proper experimental records or the reporting of results.

3)- You must start the Methods section with a paragraph headed Ethical Approval. If experiments were conducted on humans, confirmation that informed consent was obtained, preferably in writing, that the studies conformed to the standards set by the latest revision of the Declaration of Helsinki and that the procedures were approved by a properly constituted ethics committee, which should be named, must be included in the article file. If the research study was registered (clause 35 of the Declaration of Helsinki), the registration database should be indicated, otherwise the lack of registration should be noted as an exception (e.g. The study conformed to the standards set by the Declaration of Helsinki, except for registration in a database). For further information see: https://physoc.onlinelibrary.wiley.com/hub/human-experiments.

4) - Your manuscript must include a complete Additional Information section, including competing interests; funding; author contributions and acknowledgements.

5) - Please upload separate high-quality figure files via the submission form.

6) - Papers must comply with the Statistics Policy: https://jp.msubmit.net/cgi-bin/main.plex?form_type=display_requirements#statistics.

In summary:

- If n {less than or equal to} 30, all data points must be plotted in the figure in a way that reveals their range and distribution. A bar graph with data points overlaid, a box and whisker plot or a violin plot (preferably with data points included) are acceptable formats.

- If n > 30, then the entire raw dataset must be made available either as supporting information, or hosted on a not-for-profit repository, e.g. FigShare, with access details provided in the manuscript.

- 'n' clearly defined (e.g. x cells from y slices in z animals) in the Methods. Authors should be mindful of pseudoreplication.

- All relevant 'n' values must be clearly stated in the main text, figures and tables.

- The most appropriate summary statistic (e.g. mean or median and standard deviation) must be used. Standard Error of the Mean (SEM) alone is not permitted.

- Exact p values must be stated. Authors must not use 'greater than' or 'less than'. Exact p values must be stated to three significant figures even when 'no statistical significance' is claimed.

7) - Please include an Abstract Figure file and an Abstract Figure legend. An appropriate figure legend, which should not exceed 150 words in length, should be included in the main manuscript file. The Abstract Figure is a piece of artwork designed to give readers an immediate understanding of the research and should summarise the main conclusions. If possible, the image should be easily 'readable' from left to right or top to bottom. It should show the physiological relevance of the manuscript so readers can assess the importance and content of its findings. Abstract Figures should not merely recapitulate other figures in the manuscript. Please try to keep the diagram as simple as possible and without superfluous information that may distract from the main conclusion(s). Abstract Figures must be provided by authors no later than the revised manuscript stage and should be uploaded as a separate file during online submission labelled as File Type 'Abstract Figure'. Please also ensure that you include the figure legend in the main article file. All Abstract Figures should be created using BioRender. Authors should use The Journal's premium BioRender account to export high-resolution images. Details on how to use and access the premium account are included as part of this email.

8) - Please include a full title page as part of your main article (Word) file, which should contain the following: title, authors, affiliations, corresponding author name and contact details, keywords, and running title.

9) - The corresponding author must provide an institutional email address (not a personal address) for their author account. We encourage ALL co-authors to also provide institutional email addresses. If this cannot be provided (as corresponding author), then a stamped letter must be provided from the institution which confirms their role and employment there (please upload this with the revised submission).

EDITOR COMMENTS

Senior Editor:

To reiterate and accentuate a point that is included in the reviews, the burden of proof required to support the inference that changes (i.e., in the state of neural circuits) are restricted to the cortex is extremely high. Given the intrinsic limitations of the non-invasive techniques that can be applied to human volunteers, the evidence required to fully support such an inference can rarely be obtained. I would therefore urge the authors to be circumspect.

Please see the comments in the reviews concerning the presentation of individual data points, and consult the policies of the Journal.

Reviewing Editor:

This study investigated whether transcutaneous auricular vagus nerve stimulation (taVNS), when combined with a specific motor practice paradigm, boosts motor learning/performance for the hand, using a sham-controlled design. Importantly, the authors also explored some potential neurophysiological mechanisms that may underly their findings. Specifically, they used TMS of the motor cortex to map hand muscle representations and nerve stimulation to evoke F-waves, before and after the intervention, to study motor cortical and motoneuron excitability. They found that taVNS combined with motor practice increases motor cortical excitability (compared to sham with motor practice), but this doesn't drive any changes in behaviour (aka does not cause any motor learning/performance gains).

This work has been reviewed by two experts, and although they appreciated the experimental approach and overall conduct of the study, and mentioned the usefulness of findings, they identified some major concerns. Specifically, there were some questions raised about the TMS procedures and their reliability, as it is currently unclear whether the number of stimulations

used by the authors when mapping the cortex are enough to make definitive claims about corticospinal excitability and how it is affected by interventions like taVNS. Likewise, the use of F-waves instead of other approaches to assess motoneuron excitability (e.g., CMEPs, H-reflexes) needs to be better justified. There were also issues raised about data presentation, interpretation of findings, and a call for more information to better explain some parts of the manuscript text. Please see specific reviewer comments.

I believe some of these comments can be quickly addressed, but some (i.e., regarding the reliability of the TMS procedures) needs some more thought, or perhaps additional data collection.

Ethics Concerns:

In addition to the above, please mention whether the study procedures adhere to the Declaration of Helsinki.

REFEREE COMMENTS

Referee #1:

The authors present an interesting study about cortical/spinal plasticity during short-term motor practice with or without transcutaneous auricular vagus nerve stimulation. The manuscript is generally well presented, and I have provided some specific comments/suggestions below that I hope the authors find useful.

L50-53: Noted here but more generally relevant to the study - what about taVNS stimulation by itself, or prior to practice for example? Would this also be expected to have some sort of positive impact on motor recovery/neurorehabilitation? Or are effects transient enough that they are only useful when combined with motor practice?

L83-84: Why is this unclear? Seem like an important point to unpack, hence, supporting your study design.

L84-85: Do the authors suggest that this is important just for low force motor learning tasks, or also more gross high force output motor tasks?

L147: Just make sure the SHAM condition is appropriately described also.

L218: F-waves are an interesting measure, although I believe their use requires better justification over other potential measures to separate cortical and spinal components.

L228: In case I have missed it please clarify the filter settings for examining both MEPs and F-waves.

L261: Given pupil diameter is light sensitive, how did you control for this during each session and between participants?

L388: I believe it is F-wave amplitude not persistence that increased, so this should be made clear.

L403: Given this point is there a reason why a longer practice duration, or repeated sessions were not employed in this study?

L417: I would caution this statement as I feel it is very dependent on what tasks is being undertaken. For example, if we perceive early improvements in strength from motor training as a skill, then evidence does suggest adaptations occur at the spinal level.

L445: Yes, but I don't believe a strong enough rationale has been provided for the use of F-waves. Novel is sometimes good but still needs to be well justified.

L451: Caution against the word 'presented', this feels too certain given the evidence provided.

L503-510: I think this section is reasonable. However, one consideration might be what injury/impairment exists in a motor recovery setting. For example, if there are non-supraspinal problems, then based on the evidence presented here, taVNS may not be a suitable strategy. I know the evidence presented in this manuscript is not meant to be conclusive for the entire field, but this is probably worth considering and acknowledging.

Referee #2:

This study examined the locus of plasticity from a learned cyclic hand task combined with taVNS. The main finding suggests that taVNS during the task practice enhances the cortical motor representation of the APB and FDI, despite no additional behavioural benefit. The authors have completed an interesting study with a generally well-written paper. However, there are

some major and minor concerns with the methodology and reporting that should be addressed to improve the reliability and transparency of this work.

General comments:

In this study, three TMS stimuli were used to examine each point of the specified 11x11 motor grid. This approach appears problematic as MEP amplitudes are well known to be variable and small numbers of trials typically results in poor intra-session (and between-session) reliability. The representative data presented in figure 4 clearly show considerable variability in MEP size across the three stimuli at most points. The two primary measures of this experiment are the number of active points on the motor grid and the sum of mean raw MEP amplitudes across each point. It is recognized that MEPs are an inherently variable measure (particularly in resting muscles), and it is appreciated that the authors have shown transparency with raw signals. Though, this poses a critical problem for the interpretations of this data. In addition to this, there is very large variability (SD) shown for measures of active points and MEP amplitude in Figure 4 panels C and D. To achieve good to excellent reliability, a minimum of five stimuli at each point are recommended for cortical mapping using TMS (Cavaleri et al., 2017, DOI: 10.1186/s13643-017-0440-8), and both studies cited when describing the mapping procedure in this study report five stimuli at each point (Maeo et al,. 2021, DOI: 10.1111/ejn.15197, and Nakagawa et al., 2020, DOI: 10.1016/j.nicl.2019.102144). While it is understandable that measurements may need to be taken quickly given the time sensitivity of plasticity changes from a short duration intervention, limiting the number of trials is likely to have reduced the reliability of the MEP measures (map size and volume). Could the authors justify the use of few stimuli and demonstrate that the variability across three trials is not sufficient to impact measures of map size and MEP amplitude?

To improve the transparency of the results, could the authors please add the individual data to pre and post figure panels (figures 3-5) to show variability within and between participants? Additionally, for some panels (particularly Figure 4D) the error bars are hidden due to the limited y-axis range and some individual data points appear to be missing (i.e., error bars beyond individual data points). These axes should be adjusted to ensure all data are appropriately represented in the figures.

The statements (lines 451-458) suggesting that taVNS restricts plasticity changes to the cortex and not via spinal mechanisms seem overstated in its current form and require stronger support from literature. This interpretation is formed from the presented findings that 1) motor map size increased in the intervention group, and 2) there were no differences to motoneuron excitability measures in the intervention group compared to sham (i.e., sham increased following practice but not taVNS + practice). While these results could provide support for this interpretation, the underlying physiological mechanisms need to be explained and justified more clearly. For example, the vagal projections to brainstem nuclei should be described, as many brainstem systems can regulate downstream motor circuits. Additionally, this claim should be revised to reflect the brief intervention used in this study and not generalised to all taVNS interventions. Acknowledgment of possible limitations for the non-invasive methods used in this experiment should also be discussed.

Results: Effect sizes for significant results should also be reported, and for non-significant findings, specific p-values should be reported.

Specific comments:

It would be helpful for the readers to see the ball rotation task sooner in the manuscript. If possible, could the visual representation of the task shown in figure 3 be included in figure 2?

Line 194: how was the coil orientation and tilt recorded? Was this the same for post- testing?

Line 197: what was the mean MSO% for 120% RMT?

Line 204: Given that the map grid was 11x11 points, but the entire grid was not mapped, it would be helpful for the reader to see how many points were tested on average for each session.

Line 311: could the authors please report the T-statistic? This should be applied to all t-test results.

Lines 362, 364 and 366: please state the exact p-values

END OF COMMENTS

Response to Referee

EDITOR COMMENTS

Senior Editor:

To reiterate and accentuate a point that is included in the reviews, the burden of proof required to support the inference that changes (i.e., in the state of neural circuits) are restricted to the cortex is extremely high. Given the intrinsic limitations of the non-invasive techniques that can be applied to human volunteers, the evidence required to fully support such an inference can rarely be obtained. I would therefore urge the authors to be circumspect.
Please see the comments in the reviews concerning the presentation of individual data points, and consult the policies of the Journal.

Response:
We thank the Senior Editor for this important comment. In response, we have revised the manuscript to adopt a more circumspect interpretation of the data and removed statements implying that plasticity was restricted to cortical circuits. We have also clarified the limitations of the non-invasive measures used. In addition, individual data points have been added to the graphs in accordance with the Journal's policy.

Reviewing Editor:

This study investigated whether transcutaneous auricular vagus nerve stimulation (taVNS), when combined with a specific motor practice paradigm, boosts motor learning/performance for the hand, using a sham-controlled design. Importantly, the authors also explored some potential neurophysiological mechanisms that may underly their findings. Specifically, they used TMS of the motor cortex to map hand muscle representations and nerve stimulation to evoke F-waves, before and after the intervention, to study motor cortical and motoneuron excitability. They found that taVNS combined with motor practice increases motor cortical excitability (compared to sham with motor practice), but this doesn't drive any changes in behaviour (aka does not cause any motor learning/performance gains).

This work has been reviewed by two experts, and although they appreciated the experimental approach and overall conduct of the study, and mentioned the usefulness of findings, they identified some major concerns. Specifically, there were some questions raised about the TMS procedures and their reliability, as it is currently unclear whether the number of stimulations used by the authors when mapping the cortex are enough to make definitive claims about corticospinal excitability and how it is affected by interventions like taVNS. Likewise, the use of F-waves instead of other approaches to assess motoneuron excitability (e.g., CMEPs, H-reflexes) needs to be better justified. There were also issues raised about data presentation, interpretation of findings, and a call for

more information to better explain some parts of the manuscript text. Please see specific reviewer comments.

I believe some of these comments can be quickly addressed, but some (i.e., regarding the reliability of the TMS procedures) needs some more thought, or perhaps additional data collection.

Response:

We sincerely thank the Reviewing Editor for the careful evaluation of our manuscript and constructive feedback. In response to the concerns raised, we have substantially revised the manuscript to address the main concerns regarding the reliability of the TMS mapping procedures, the rationale for using F-wave measurements, and the presentation and interpretation of the data. The major revisions are summarised below.

1) To address the Reviewing Editor's concern regarding the reliability of TMS mapping using three stimuli per grid point, we conducted an additional validation experiment directly comparing mapping outcomes obtained with three versus five stimuli per grid point under identical experimental conditions. The results demonstrated good agreement between the estimates obtained with the two protocols in Bland–Altman analyses, indicating that the use of three stimuli per site provides comparable estimates of map size and map volume to those obtained with five stimuli. These additional analyses support the reliability of the mapping outcomes obtained with three stimuli per grid point and directly address the concern raised by the reviewers and the Reviewing Editor regarding the adequacy of the stimulation number. These validation results have been incorporated into the **Supplementary Information**. We have also clarified the rationale for using three stimuli per grid point in the revised manuscript **(Lines 223-)** and acknowledged this methodological choice as a consideration and potential limitation **(Lines 536-)**.

2) We agree that the rationale for using F-wave measurements required clearer explanation. Accordingly, we have revised the manuscript to clarify the physiological interpretation of F-waves as an index of spinal motoneuron pool excitability and to explain why this measure was selected **(Lines 80-)**. Specifically, other commonly used measures of spinal excitability involve additional neural components and are therefore less specific to the motoneuron pool excitability. For example, the H-reflex depends on Ia afferent transmission and spinal interneuronal processing, while cervicomedullary motor evoked potentials (CMEPs) reflect transmission along corticospinal pathways. Because the present study aimed to evaluate changes at the level of the motoneuron pool, we selected F-wave measurements as an appropriate index.

3) Regarding data presentation and interpretation, we have added individual data points to the graphs and specific statistical values (exact p-value [including non-significant results], t-value, effect size, and related statistics). We have adapted a more circumspect interpretation of the

data.

Ethics Concerns:

In addition to the above, please mention whether the study procedures adhere to the Declaration of Helsinki.

Response:

Thank you. We have added a statement clarifying that the study procedures adhere to the Declaration of Helsinki.

Change [Lines 121-]:

Ethical approval

This study was approved by the Human Research Ethics Committee of Waseda University (approval number: 2020-93). Written informed consent was obtained from all participants. The study conformed to the standards set by the Declaration of Helsinki, except for registration in a database because the study was not a clinical trial.

Referee #1:

The authors present an interesting study about cortical/spinal plasticity during short-term motor practice with or without transcutaneous auricular vagus nerve stimulation. The manuscript is generally well presented, and I have provided some specific comments/suggestions below that I hope the authors find useful.

L50-53: Noted here but more generally relevant to the study - what about taVNS stimulation by itself, or prior to practice for example? Would this also be expected to have some sort of positive impact on motor recovery/neurorehabilitation? Or are effects transient enough that they are only useful when combined with motor practice?

Response:

We thank the reviewer for raising this important point. As discussed in the Introduction, previous studies have reported beneficial effects of taVNS delivered at different timings relative to motor training, including stimulation paired with discrete movements as well as continuous stimulation applied at rest before or during rehabilitation therapy (e.g., Wu et al., 2020; Li et al., 2022). These findings suggest that the optimal timing of taVNS relative to motor practice remains unresolved.

In the present study, our aim was specifically to examine how taVNS delivered during ongoing motor practice influences the expression of practice-related plasticity within the motor system. Therefore, we focused on the pairing of taVNS with active motor practice rather than including a taVNS-only condition. We agree that investigating the effects of taVNS delivered alone or prior to practice would be an important direction for future studies.

We have revised the sentence in the Abstract to clarify that the interpretation specifically refers to taVNS paired with motor practice, thereby avoiding overgeneralization.

Change [Lines 50-]:

By relating taVNS-induced plasticity to noradrenergic engagement and revealing differential cortical and spinal responses, this study offers novel mechanistic insights into how pairing taVNS with motor training may establish a neural basis for motor recovery.

L83-84: Why is this unclear? Seem like an important point to unpack, hence, supporting your study design.

Response:

We thank the reviewer for this comment. We agree that the rationale for this statement requires clarification. Most evidence linking taVNS to LC–noradrenergic activation has been obtained under resting conditions. Because engagement in motor behavior itself modulates arousal and noradrenergic

activity, the interaction between taVNS and ongoing motor behavior remains uncertain. We have revised the text to clarify this point.

Change [Lines 90-]:

However, most evidence linking taVNS to LC–NA activation has been obtained under resting conditions. Because engagement in motor behaviour itself modulates arousal and noradrenergic activity (Aston-Jones and Cohen, 2005), it remains unclear whether taVNS delivered during ongoing motor behaviour engages similar neuromodulatory mechanisms. Monitoring pupil dynamics during taVNS paired with motor learning may therefore provide a useful window into how vagal stimulation interacts with task-related noradrenergic modulation during motor behaviour.

L84-85: Do the authors suggest that this is important just for low force motor learning tasks, or also more gross high force output motor tasks?

Response:

We thank the reviewer for this insightful comment. The present study employed a fine motor learning task involving sequential finger movements, which typically require relatively low force output. Our results therefore do not allow conclusions about tasks requiring higher force production. As discussed in the manuscript **(Lines 505-)**, the effectiveness of taVNS may depend on the type of motor learning and the underlying locus of plasticity (e.g., cortical/cerebellar vs spinal mechanisms). Future studies should therefore examine a broader range of motor tasks to determine which types of motor learning are most susceptible to taVNS-induced behavioural enhancement. In this revision, we have specified that the present findings pertain to “dexterous” motor practice to avoid overgeneralization.

Change [Lines 22-]:

This study shows that, in healthy adults, taVNS paired with dexterous motor practice enhanced cortical plasticity (increased M1 map size) without immediate behavioural improvement.

Change [Lines 38-]:

Here, we demonstrate that taVNS paired with dexterous motor skill practice selectively enhances cortical plasticity without boosting motor performance during short-term training.

Change [Lines 94-]:

Monitoring pupil dynamics during taVNS paired with dexterous motor learning may therefore provide a useful window into how vagal stimulation interacts with task-related noradrenergic modulation during motor behaviour.

Change [Lines 113-]:

We investigated the effects of taVNS paired with periodic dexterous motor skill practice on behavioural performance and plasticity in motor-related neural circuits.

Change [Lines 512-]:

Therefore, the expression of plasticity in the present task may have been biased toward supraspinal circuits, which could partly explain the limited behavioural benefit observed in this task.

Change [Lines 629-]:

In summary, taVNS paired with dexterous motor practice did not enhance motor learning in the short term but selectively promoted reorganization of the M1 map without detectable spinal plastic change.

L147: Just make sure the SHAM condition is appropriately described also.

Response:

Thank you for this comment. In this revision, we now clarify that no stimulation intensity adjustment was performed in the sham condition, as no electrical current was delivered.

Change [Line 173]:

Therefore, no intensity adjustment was performed in the sham group.

L218: F-waves are an interesting measure, although I believe their use requires better justification over other potential measures to separate cortical and spinal components.

L445: Yes, but I don't believe a strong enough rationale has been provided for the use of F-waves. Novel is sometimes good but still needs to be well justified.

Response:

We thank the reviewer for raising this important point regarding the rationale for using F-wave measurements. In the present study, our aim was to distinguish potential plastic changes occurring at the cortical level (assessed by TMS motor mapping) from those occurring at the level of the spinal motoneuron pool. F-waves are widely used as an index of motoneuron pool excitability because they reflect antidromic activation and backfiring of α-motoneurons.

Other commonly used measures of spinal excitability involve additional neural components. For example, the H-reflex depends on Ia afferent transmission and spinal interneuronal processing, while cervicomedullary motor evoked potentials (CMEPs) reflect transmission along corticospinal pathways. Because the present study specifically aimed to evaluate potential changes at the level of the motoneuron pool, we selected F-wave measurements as an appropriate index.

We agree that the rationale for using F-wave measurements required clearer explanation. Accordingly, we have revised the manuscript to clarify the physiological interpretation of F-waves as an index of spinal

motoneuron pool excitability and to explain why this measure was selected. In addition, we have softened the wording regarding novelty.

Change [Lines 80-]:

To isolate potential changes at the level of the motoneuron pool with minimal contributions from afferent pathways or descending corticospinal drive, we assessed F-waves as an index of spinal motoneuron excitability. F-waves primarily reflect motoneuron excitability through antidromic activation and backfiring of α-motoneurons (Fisher, 1992). To our knowledge, the impact of taVNS on spinal motoneuron excitability has not been systematically examined. Furthermore, this approach allowed us to examine plastic changes at both cortical and spinal levels within the same experimental framework.

Change [Lines 239-]:

F-waves are widely used as an index of spinal motoneuron pool excitability because they reflect antidromic activation and backfiring of α-motoneurons without involving synaptic transmission through sensory afferents.

Change [Lines 548-]:

To explore plasticity beyond the brain, we measured F-waves as an index of spinal motoneuron excitability. This approach enabled us to examine plastic changes at both cortical and spinal levels within the same experimental framework and provides insight into the hierarchical expression of plasticity in the motor system.

L228: In case I have missed it please clarify the filter settings for examining both MEPs and F-waves.

Response:

Thank you for this comment. The EMG recording and filtering settings were identical for both MEP and F-wave measurements. We have clarified this point in the Methods section by stating that the same EMG acquisition parameters (band-pass filter 5–1500 Hz) were used for both measurements.

Change [Lines 209-]:

The same EMG recording and filtering settings were used for both MEP and F-wave measurements.

L261: Given pupil diameter is light sensitive, how did you control for this during each session and between participants?

Response:

Thank you for this important point. All pupil recordings were conducted in the same experimental room under stable artificial lighting conditions. No external light sources (e.g., sunlight or light from adjacent rooms) entered the room, and the lighting conditions were kept constant across all sessions and participants. We have now clarified this point in the Methods section.

Change [Lines 278-]:

All recordings were conducted in the same experimental room under stable artificial lighting conditions with no external light sources, ensuring consistent illumination across sessions and participants.

L388: I believe it is F-wave amplitude not persistence that increased, so this should be made clear.

Response:

Thank you for this helpful comment. We agree that the specific F-wave parameter showing change should be clearly stated. We have revised the sentence in the Discussion to specify that F-wave amplitude increased in the sham group, and clarified its interpretation in relation to spinal motoneuron excitability.

Change [Lines 473-]:

F-wave amplitude increased in the sham group but not in the taVNS group, consistent with increased spinal motoneuron excitability following motor practice in the absence of stimulation.

L403: Given this point is there a reason why a longer practice duration, or repeated sessions were not employed in this study?

Response:

We appreciate the reviewer for this comment. The aim of the present study was not to evaluate the rehabilitative efficacy of taVNS through prolonged training, but rather to use a controlled motor-learning paradigm as an experimental model to investigate potential neural mechanisms that may contribute to taVNS-facilitated motor performance. Therefore, we employed a short-term practice protocol paired with stimulation to examine early neurophysiological responses. We agree that longer practice durations or repeated sessions may be necessary to reveal behavioural benefits, as suggested by clinical studies using multi-day or multi-week interventions. We have clarified this point in the Discussion.

Change [Lines 488-]:

Longer interventions may reveal behavioral benefits, as many studies demonstrating functional improvements with VNS have employed training protocols lasting from days to weeks (Gerges et al., 2024).

L417: I would caution this statement as I feel it is very dependent on what tasks is being undertaken. For example, if we perceive early improvements in strength from motor training as a skill, then evidence does suggest adaptations occur at the spinal level.

Response:

Thank you for this important comment. We agree that the neural substrates of motor learning are task-dependent. To clarify this point, we have revised the statement in the Discussion to avoid overgeneralization and to acknowledge that the contribution of spinal circuits may vary depending on the task and training conditions.

Change [Lines 505-]:

Motor adaptation has been thought to involve primarily cerebellar and cortical mechanisms, although the relative contribution of spinal circuits may vary depending on the specific task and training conditions (Ahmadi-Pajouh et al., 2012; Ito et al., 2021).

L451: Caution against the word 'presented', this feels too certain given the evidence provided.

Response:

Thank you for this comment. We agree that our previous wording may have overstated the interpretation that taVNS “prevent” the spinal changes. In the revised manuscript, we have softened the language to avoid implying that taVNS prevents spinal plasticity.

Change [Lines 555-]:

In contrast, such spinal-level changes were not observed when practice was paired with taVNS. These findings indicate that the locus at which practice-related plasticity was expressed differed between conditions.

Change [Lines 568-]:

That is, motor practice alone in this study may lead to measurable adaptation at the level of the motoneuron pool, whereas pairing practice with taVNS may have been associated with a relative shift in the measurable expression of plasticity toward supraspinal circuits.

L503-510: I think this section is reasonable. However, one consideration might be what injury/impairment exists in a motor recovery setting. For example, if there are non-supraspinal problems, then based on the evidence presented here, taVNS may not be a suitable strategy. I know the evidence presented in this manuscript is not meant to be conclusive for the entire field, but this is probably worth considering and acknowledging.

Response:

Thank you for this important comment. We agree that the therapeutic implications of taVNS may depend on the locus of neural dysfunction. To acknowledge this point, we have added a statement in the Discussion noting that if taVNS preferentially facilitates cortical plasticity, its clinical effectiveness may vary depending on whether the primary pathology is supraspinal or spinal. We further note that many

reported clinical applications of taVNS involve conditions such as stroke, whereas its potential relevance for disorders primarily affecting spinal circuits remains uncertain.

Change [Lines 578-]:

If taVNS preferentially facilitates cortical plasticity, its therapeutic potential may depend on the locus of neural dysfunction. This may partly explain why several clinical studies reporting beneficial effects of taVNS have been reported in conditions involving supraspinal dysfunction (e.g., stroke) (Ahmed et al., 2023; Gerges et al., 2024), although further work is needed to determine whether similar benefits extend to disorders primarily involving spinal pathology.

Referee #2:

This study examined the locus of plasticity from a learned cyclic hand task combined with taVNS. The main finding suggests that taVNS during the task practice enhances the cortical motor representation of the APB and FDI, despite no additional behavioural benefit. The authors have completed an interesting study with a generally well-written paper. However, there are some major and minor concerns with the methodology and reporting that should be addressed to improve the reliability and transparency of this work.

General comments:

In this study, three TMS stimuli were used to examine each point of the specified 11x11 motor grid. This approach appears problematic as MEP amplitudes are well known to be variable and small numbers of trials typically results in poor intra-session (and between-session) reliability. The representative data presented in figure 4 clearly show considerable variability in MEP size across the three stimuli at most points. The two primary measures of this experiment are the number of active points on the motor grid and the sum of mean raw MEP amplitudes across each point. It is recognized that MEPs are an inherently variable measure (particularly in resting muscles), and it is appreciated that the authors have shown transparency with raw signals. Though, this poses a critical problem for the interpretations of this data. In addition to this, there is very large variability (SD) shown for measures of active points and MEP amplitude in Figure 4 panels C and D. To achieve good to excellent reliability, a minimum of five stimuli at each point are recommended for cortical mapping using TMS (Cavaleri et al., 2017, DOI: 10.1186/s13643-017-0440-8), and both studies cited when describing the mapping procedure in this study report five stimuli at each point (Maeo et al,. 2021, DOI: 10.1111/ejn.15197, and Nakagawa et al., 2020, DOI: 10.1016/j.nicl.2019.102144). While it is understandable that measurements may need to be taken quickly given the time sensitivity of plasticity changes from a short duration intervention, limiting the number of trials is likely to have reduced the reliability of the MEP measures (map size and volume). Could the authors justify the use of few stimuli and demonstrate that the variability across three trials is not sufficient to impact measures of map size and MEP amplitude?

Response:

We thank the reviewer for this thoughtful and important comment regarding the number of stimuli per grid point and the potential impact of MEP variability on the reliability of our mapping outcomes.

We fully acknowledge that previous methodological studies have generally recommended the use of at least five stimuli per site to achieve good-to-excellent reliability in TMS mapping (e.g., Cavaleri et al., 2017), and we were aware of these recommendations when designing the present study. However, our primary concern was the potential time sensitivity of neuroplastic changes induced by the combined vagus nerve stimulation and motor training intervention. As the duration and temporal stability of the induced plasticity were unknown, we prioritised minimizing post-intervention assessment time in order to reduce the risk of underestimating transient physiological effects. Consequently, three stimuli per grid

point were used to shorten total mapping duration while maintaining systematic cortical sampling.

To directly address the reviewer's concern regarding reliability, we conducted an additional validation experiment in an independent sample (N = 10), directly comparing mapping outcomes derived from three versus five stimuli per grid point under identical experimental conditions. The results of the validation experiment are shown in Figure S1. Pearson correlation coefficients ranged from r = 0.79 to r = 0.98 (mean r = 0.91), indicating strong linear associations between the two protocols across both muscles and outcome measures (map size and map volume) (Fig. S1A, C, E, and G). Importantly, Bland–Altman analyses revealed minimal systematic bias and narrow limits of agreement (Fig. S1B, D, F, and H), supporting good agreement between the two protocols. Therefore, estimates obtained with three stimuli per grid point provide comparable mapping outcomes to the conventional five-stimulus protocol under the present mapping conditions. We have incorporated this validation analysis into the Supplementary Information and clarified the rationale for using three stimuli per grid point in the revised manuscript.

Meanwhile, we agree that increasing the number of stimuli per site may further enhance measurement stability. We have therefore added a statement in the revised manuscript acknowledging the use of three stimuli per grid point as a methodological consideration and discussing it as a limitation. This revision clarifies that the choice reflects a trade-off between measurement precision and temporal sensitivity of the intervention effects, rather than an oversight of established methodological recommendations.

We hope this additional validation analysis and clarification sufficiently address the reviewer's concern.

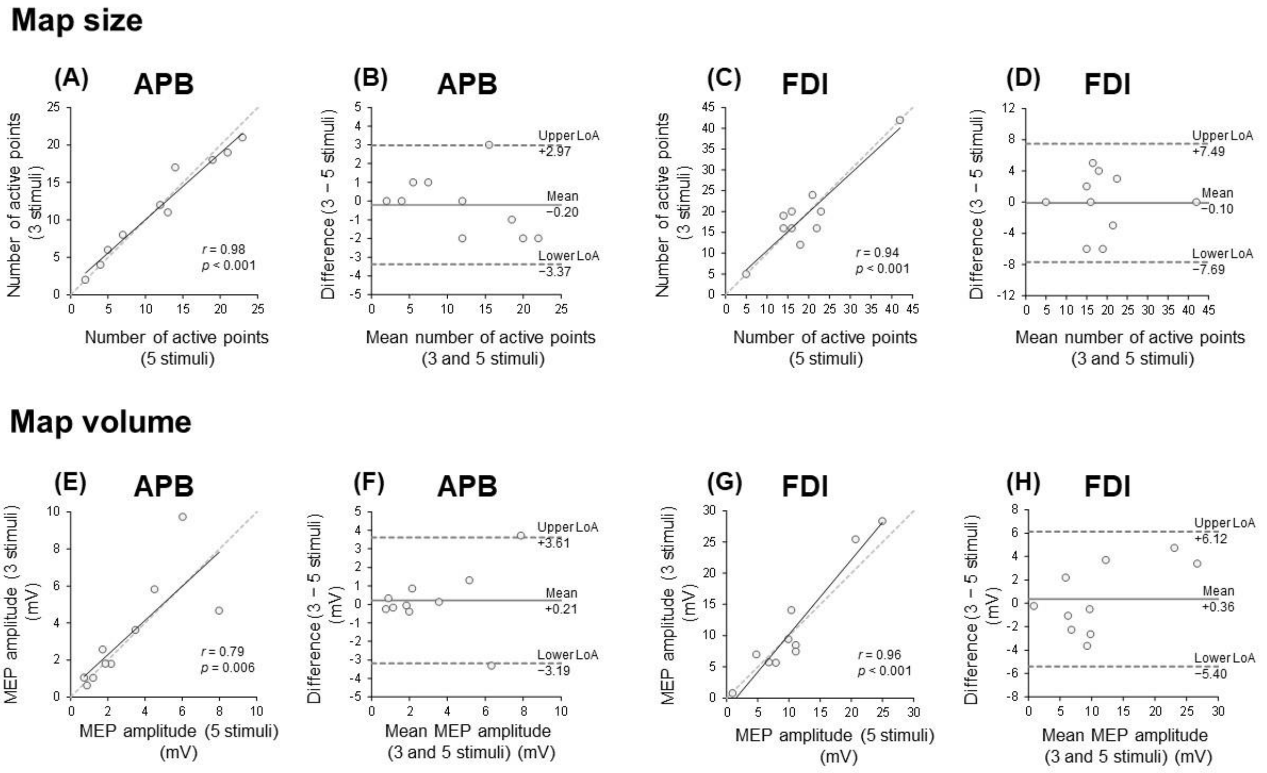

Figure S1. Validation of TMS mapping outcomes obtained with 3 versus 5 stimuli per grid point in APB and FDI muscles

Change [Lines 223-]:

This number of stimuli was chosen to minimise the duration of post-intervention assessment given the uncertain temporal stability of the induced plasticity. A validation analysis comparing three versus five stimuli per grid point is provided in the Supplementary Information.

Change [Lines 536-]:

One methodological consideration relates to the number of TMS stimuli delivered at each grid point during motor mapping. In the present study, three stimuli were used per site to minimise the duration of post-intervention assessment because the temporal stability of taVNS-induced plasticity following short-term training was unknown. Although previous studies have recommended a minimum of five stimuli to maximise mapping reliability (Cavaleri et al., 2017), an additional validation experiment demonstrated strong agreement between three- and five-stimulus protocols under the present mapping conditions (see Supplementary Information). Importantly, the primary mapping outcomes analysed in this study (map size and map volume) represent aggregated measures derived across multiple grid points rather than single-trial MEP amplitudes. Nevertheless, it remains possible that a larger number of stimuli could further improve measurement stability.

To improve the transparency of the results, could the authors please add the individual data to pre and post figure panels (figures 3-5) to show variability within and between participants? Additionally, for some panels (particularly Figure 4D) the error bars are hidden due to the limited y-axis range and some individual data points appear to be missing (i.e., error bars beyond individual data points). These axes should be adjusted to ensure all data are appropriately represented in the figures.

Response:

We thank the reviewer for this suggestion. Individual participant data have now been added to all Pre–Post panels (Figures 3–5), in accordance with Journal guidelines. The y-axis ranges have also been adjusted to ensure that all individual data points and the error bars are fully visible. We believe that these changes improve transparency and allow clearer visualisation of within- and between-participant variability.

The statements (lines 451-458) suggesting that taVNS restricts plasticity changes to the cortex and not via spinal mechanisms seem overstated in its current form and require stronger support from literature. This interpretation is formed from the presented findings that 1) motor map size increased in the intervention group, and 2) there were no differences to motoneuron excitability measures in the intervention group compared to sham (i.e., sham increased following practice but not taVNS + practice). While these results could provide support for this interpretation, the underlying physiological mechanisms need to be explained and justified more clearly. For example, the vagal projections to brainstem nuclei should be described, as many brainstem systems can regulate downstream motor circuits. Additionally, this claim should be revised to reflect the brief intervention used in this study and not generalised to all taVNS interventions. Acknowledgment of possible limitations for the non-invasive methods used in this experiment should also be discussed.

Response:

We thank the reviewer for this insightful comment. We agree that our previous wording may have overstated the interpretation that taVNS "restricts" plasticity to cortical circuits, and we have substantially revised this section to provide a more mechanistic and balanced discussion.

First, we have softened the language to avoid implying that taVNS prevents spinal plasticity. Instead, we now state that, within the present short-term intervention, taVNS was associated with cortical reorganization without detectable changes in F-wave indices. We further clarify that this pattern may reflect a relative shift in the measurable expression of plasticity rather than an absolute suppression of spinal neural circuits.

Second, in response to the reviewer's request for clearer physiological justification, we have expanded the description of vagal projections and their relevance to motor control. Specifically, we now describe how vagal afferents project to the nucleus of the solitary tract (NTS), which in turn innervates neuromodulatory nuclei such as the locus coeruleus (LC) and basal forebrain (Rodenkirch et al., 2022). We also note that brainstem nuclei including the LC and pontomedullary reticular formation possess descending projections capable of influencing spinal motoneurons (Proudfit & Clark, 1991; Brownstone & Chopek, 2018). These additions clarify that taVNS has the anatomical potential to influence multiple hierarchical levels of the motor system.

Third, we have explicitly limited our interpretation to the short-term protocol used in this study and clarified that the proposed hierarchical shift in plasticity expression may not extend to other taVNS paradigms.

Finally, we now explicitly acknowledge the methodological limitations of the non-invasive measures used in this study. F-wave measurements provide an indirect and selective index of spinal motoneuron excitability and may not capture all forms of spinal plasticity. Similarly, TMS motor mapping provides an indirect index of cortical representational organization and cannot directly specify the underlying synaptic or circuit-level mechanisms. Accordingly, we clarify that conclusions regarding the hierarchical locus of plasticity should be interpreted with caution and that additional spinal mechanisms cannot be excluded.

We believe these revisions address the reviewer's concerns by (1) tempering the claim, (2) providing clearer anatomical and physiological context, (3) limiting generalization, and (4) acknowledging methodological constraints.

Change [Lines 24]:

By contrast, sham stimulation alone increased F-wave amplitude, suggesting that taVNS may promote a relative shift in the expression of plasticity toward cortical circuits.

Change [Lines 45-]:

F-wave amplitude increased in the sham group but not in the taVNS group, suggesting a relative shift in the expression

of practice-related plasticity toward cortical circuits when training was paired with taVNS. This pattern suggests that taVNS may influence the hierarchical expression of motor plasticity.

Change [Lines 555-]:

In contrast, such spinal-level changes were not observed when practice was paired with taVNS. These findings indicate that the locus at which practice-related plasticity was expressed differed between conditions.

Vagal afferents project to the nucleus of the solitary tract (NTS), which innervates multiple brainstem nuclei including the locus coeruleus (LC) and basal forebrain (Rodenkirch et al., 2022), thereby modulating cortical plasticity via ascending neuromodulatory systems. At the same time, brainstem nuclei such as LC and pontomedullary reticular formation possess descending projections capable of influencing spinal motoneuron (Proudfit and Clark, 1991; Brownstone and Chopek, 2018). Thus, anatomically, taVNS has the potential to modulate plasticity at multiple hierarchical levels of the motor system.

However, under the present short-term intervention we observed cortical reorganization without detectable changes in F-wave indices in the taVNS group. Taken together, these results suggest that neuromodulatory context may influence the level at which expression of plasticity is expressed within the motor system. That is, motor practice alone in this study may lead to measurable adaptation at the level of the motoneuron pool, whereas pairing practice with taVNS may have been associated with a relative shift in the measurable expression of plasticity toward supraspinal circuits. This interpretation regarding the shift in the measurable expression of plasticity should be considered in the context of the brief intervention used in the present study and may not extend to other taVNS paradigms. We do not exclude the possibility that additional spinal mechanisms were engaged but not captured by F-wave measurements. Furthermore, it should be noted that both TMS mapping and F-wave measurements provide indirect indices of neural plasticity and cannot definitively localise the underlying synaptic mechanisms. Therefore, conclusions regarding the hierarchical locus of plasticity should be interpreted with appropriate caution.

Results: Effect sizes for significant results should also be reported, and for non-significant findings, specific p-values should be reported.

Response:

We thank the reviewer for this important comment. We have revised the Results section to improve statistical transparency. Specifically, we now report:

(1) effect sizes (Cohen's *d* for t-tests, *r* for non-parametric tests, and η_p^2 for ANOVA),

(2) exact p-values for all non-significant results, and

(3) the corresponding test statistics (t, or Z value, as appropriate) for each analysis.

These additions have been implemented consistently throughout the Results section.

Specific comments:

It would be helpful for the readers to see the ball rotation task sooner in the manuscript. If possible, could the visual representation of the task shown in figure 3 be included in figure 2?

Response:
Thank you for this helpful comment. To improve the clarity and flow of the manuscript, we have moved the visual representation of the ball-rotation task from Figure 3 to Figure 2, where the overall experimental protocol is illustrated. This allows readers to understand the motor task earlier in the manuscript.

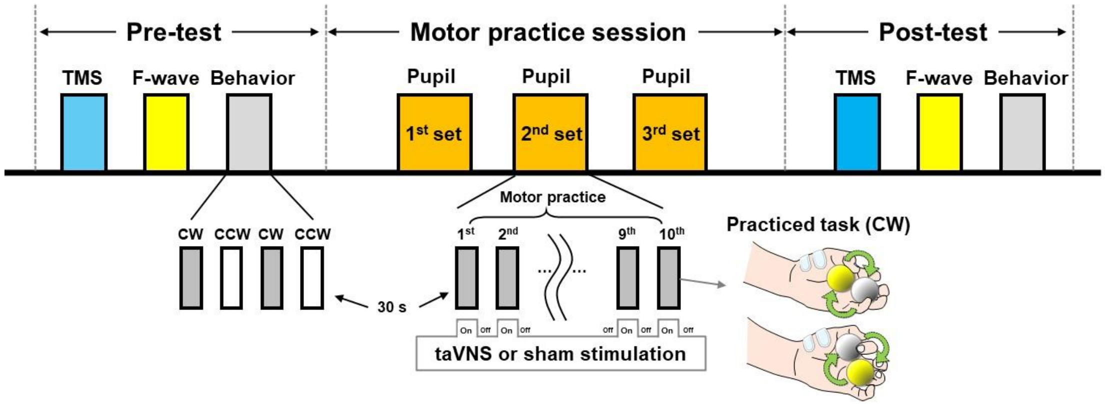

Revised Figure 2

Line 194: how was the coil orientation and tilt recorded? Was this the same for post- testing?

Response:
Thank you for this important clarification. We apologise for the inaccurate wording in the original manuscript. During motor mapping, the spatial position of the coil was guided using the Brainsight neuronavigation system; however, coil orientation and tilt were manually maintained by the experimenter and were not digitally recorded. The same procedure was used for both Pre- and Post-testing sessions. We have revised the manuscript accordingly to clarify this point and removed the statement suggesting that orientation and tilt were recorded.

Change [Line 219]:
Coil position was continuously monitored with a navigation system (Brainsight version 2.2.8, Rogue Research, Montreal, Canada).

Change [Line 229]:
The same procedure was applied during both pre- and post-testing sessions.

Line 197: what was the mean MSO% for 120% RMT?

Response:

Thank you for this comment. We have now added the mean ± SD stimulation intensity expressed as percentage of maximal stimulator output (MSO) corresponding to 120% RMT for each group.

Change [Lines 217-]:

Motor maps were then obtained using TMS at 120% RMT (taVNS: 61.3 ± 10.4 % of maximal stimulator output (MSO), sham: 58.9 ± 11.9 % MSO) within an 11×11 cm grid (1 cm spacing) centered on the hotspot (Figure 4A).

Line 204: Given that the map grid was 11x11 points, but the entire grid was not mapped, it would be helpful for the reader to see how many points were tested on average for each session.

Response:

We agree that reporting the number of stimulated grid points increases transparency. We have now reported the mean ± SD number of stimulated grid points for each group and session in the revised manuscript.

Change [Lines 346-]:

The number of stimulated grid points was 42.9 ± 11.3 in the taVNS_Pre, 47.3 ± 14.7 in the taVNS_Post, 47.1 ± 10.9 in the sham_Pre, and 42.1 ± 11.9 in the sham_Post.

Line 311: could the authors please report the T-statistic? This should be applied to all t-test results.

Lines 362, 364 and 366: please state the exact p-values

Response:

We thank the reviewer for this important comment. We have revised the Results section to improve statistical transparency. Specifically, we now report:

(1) effect sizes (Cohen's *d* for t-tests, *r* for non-parametric tests, and η_p^2 for ANOVA),

(2) exact p-values for all non-significant results, and

(3) the corresponding test statistics (t, or Z value, as appropriate) for each analysis.

These additions have been implemented consistently throughout the Results section.

END OF COMMENTS

Dear Dr Nakagawa,

Re: JP-RP-2026-290398R1 "**Transcutaneous auricular vagus nerve stimulation during short-term motor practice drives cortical plasticity without behavioural improvement** " by Kento Nakagawa and Rieko Osu

Thank you for submitting your manuscript to The Journal of Physiology. It has been assessed by a Reviewing Editor and by 2 expert referees and we are pleased to tell you that it is acceptable for publication following satisfactory revision.

The referee reports are copied at the end of this email.

Please address all the points raised and incorporate all requested revisions or explain in your Response to Referees why a change has not been made. We hope you will find the comments helpful and that you will be able to return your revised manuscript within 4 weeks. If you require longer than this, please contact journal staff: jp@physoc.org.

Your revised manuscript should be submitted online using the link in your Author Tasks Link Not Available. This link is accessible via your account as Corresponding Author; it is not available to your co-authors. If this presents a problem, please contact journal staff (jp@physoc.org). Image files from the previous version are retained on the system. Please ensure you replace or remove any files that are being revised.

If you do not wish to submit a revised version of your manuscript, you must inform our journal staff (jp@physoc.org) or reply to this email to request withdrawal. Please note that a manuscript must be formally withdrawn from the peer review process at one journal before it may be submitted to another journal.

TRANSPARENT PEER REVIEW POLICY: To improve the transparency of its peer review process The Journal of Physiology publishes online, as supporting information, the peer review history of all articles accepted for publication. Readers will have access to decision letters, including Editors' comments and referee reports, for each version of the manuscript, as well as any author responses to peer review comments. Referees can decide whether or not they wish to be named on the peer review history document.

ABSTRACT FIGURES: Authors are expected to use The Journal's premium BioRender account to create/redraw their Abstract Figures. Information on how to access this account is here: https://physoc.onlinelibrary.wiley.com/journal/14697793/biorender-access.

This will enable Authors to create and download high-resolution figures. If authors have used the free BioRender service, they can use the instructions provided in the link above to download a high-resolution version suitable for publication.

The link provided should only be used for the purposes of this submission. Authors will be charged for figures created on this account if they are not related to this manuscript submission.

LANGUAGE EDITING AND SUPPORT FOR PUBLICATION: If you would like help with English language editing, or other article preparation support, Wiley Editing Services offers expert help, including English Language Editing, as well as translation, manuscript formatting, and figure formatting at www.wileyauthors.com/eeo/preparation. You can also find resources for Preparing Your Article for general guidance about writing and preparing your manuscript at www.wileyauthors.com/eeo/prepresources.

RIGOUR AND REPRODUCIBILITY: We recommend authors consult The Journal's Rigour and Reproducibility requirements to ensure that their manuscript is suitable for publication: https://physoc.onlinelibrary.wiley.com/pb-assets/hub-assets/physoc/documents/TJP-Rigour-and-Reproducibility-Requirements.pdf

REVISION CHECKLIST:

Check that your Methods section conforms to journal policy: https://jp.msubmit.net/cgi-bin/main.plex?form_type=display_requirements#methods

Check that data presented conforms to the statistics policy: https://jp.msubmit.net/cgi-bin/main.plex?form_type=display_requirements#statistics

Upload a full Response to Referees file. To create your 'Response to Referees' copy all the reports, including any comments from the Senior and Reviewing Editors, into a Microsoft Word, or similar, file and respond to each point, using font or background colour to distinguish comments and responses and upload as the required file type.

Please upload two versions of your manuscript text: one with all relevant changes highlighted and one clean version with no changes tracked. The manuscript file should include all tables and figure legends, but each figure/graph should be uploaded as separate, high-resolution files. The journal is now integrated with Wiley's Image Checking service. For further details, see: https://www.wiley.com/en-us/network/publishing/research-publishing/trending-stories/upholding-image-integrity-wileys-image-screening-service

You may also upload:

- 'Potential Cover Art' for consideration as the issue's cover image
- Appropriate Supporting Information (Video, audio or data set: see https://jp.msubmit.net/cgi-bin/main.plex?form_type=display_requirements#supp).

We look forward to receiving your revised submission.

If you have any queries, please reply to this email and we will be pleased to advise.

Yours sincerely,

Richard Carson
Senior Editor
The Journal of Physiology

EDITOR COMMENTS

Reviewing Editor:

I commend the authors on addressing and responding to the comments, queries and concerns raised by the two expert referees, and updating the manuscript accordingly. Both referees have raised some additional considerations that need to be addressed.

Senior Editor:

Comments for Authors to ensure the paper complies with the Statistics Policy:
Please note that exact p values must be given in all instances. In the present version of the manuscript, statements such as ($p < .05$) are included (e.g., in the Pupil dilation section).

Comments to the Author:
Please note that the Journal does not permit the inclusion of substantive content as "Supplementary Information". All relevant information should be included in the main body of the manuscript. As there is no word limit, this requirement should not pose a challenge. The "Supporting Information for online publication" should be used only for "Data set/Video/Audio only".

REFEREE COMMENTS

Referee #1:

The authors have suitably addressed most of my original comments. I do, however, have a couple of additional comments based on the responses provided.

Regarding the controlling of light levels within the experimental setting...I cannot see raw data for pupil diameter presented anywhere (unless I have missed it). Simply, showing these values to the similar at baseline (not normalised to 100%) would give more confidence in the rebuttal of my initial comment about light levels impacting pupil diameter, and therefore, more confidence in the between condition results.

For MEP and F-wave filtering...I appreciate the additional information although I do wonder why the same filtering was applied for both? Specifically, I understand the high-pass filter setting to help with F-waves (although some studies use even lower), but this seems a little low based on multiple studies of MEP recordings. E.g., Some evidence suggest that high-pass filters set too low can impact measured MEP characteristics: https://pmc.ncbi.nlm.nih.gov/articles/PMC8469458/. Is there any reason why two separate filters were not applied for each measurement. Or additional filtering for F-waves after collection of the initial signals?

Referee #2:

The authors have significantly enhanced the transparency of the study, and addressed most prior concerns appropriately. It is appreciated that the authors have collected additional data to demonstrate the reliability of the study, and this adds additional support for the methods used. There are some minor comments listed below to further enhance the transparency of the manuscript.

Line 219 to 222: While it is appreciated that the authors have now clarified how the coil position was monitored, there should still be mention of how tilt and orientation was managed and kept consistent (i.e., whether the same or different experimenters performed TMS across different sessions).

Line 560 to 562: the effects of the descending projections on spinal motoneurons is not clear based on this sentence alone, particularly when considering the function of the reticular formation. Could the authors rephrase or add an additional statement to indicate how, or to what extent this may influence spinal motoneuron activity related to motor learning of such voluntary tasks? This will make the physiological underpinning of the possible plasticity effects more apparent to the reader.

Figure 7: Additional detail should be added to the figure caption to improve the clarity of the proposed plasticity pathway/effects represented in this figure. While the figure itself is well presented, a more detailed description to provide necessary supporting information will improve the interpretability.

END OF COMMENTS

EDITOR COMMENTS

Reviewing Editor:

I commend the authors on addressing and responding to the comments, queries and concerns raised by the two expert referees, and updating the manuscript accordingly. Both referees have raised some additional considerations that need to be addressed.

Response:

We sincerely thank the Reviewing Editor for the careful evaluation of our manuscript. We have revised the manuscript to address the concerns from the editors and reviewers.

Senior Editor:

Comments for Authors to ensure the paper complies with the Statistics Policy:
Please note that exact p values must be given in all instances. In the present version of the manuscript, statements such as ($p < .05$) are included (e.g., in the Pupil dilation section).

Response:

We thank the Senior Editor for this important comment. In response, we re-analysed the pupil time-series data and calculated exact cluster-level p values for the significant clusters. These values are now reported in the revised manuscript in accordance with the journal's statistical reporting guidelines. This analysis yielded results consistent with our original findings, with significant clusters observed in similar time intervals. These changes have been incorporated into the Results section and Figure 6.

Change [lines 439-]:

Pupil dilation (Fig. 6A)

Figure 6A shows the normalised pupil diameter from the onset of task/rest periods (0 s) to 30 s. During the task period, the taVNS group exhibited a significant pupil dilation relative to baseline between 0.2–2.3 s (cluster $p = 0.0298$), whereas no significant change was observed in the sham group. During the rest period, both groups showed significant pupil constriction relative to baseline: 1.2–25.0 s for taVNS (cluster $p < 0.001$) and 2.8–25.0 s for sham group (cluster $p = 0.0028$). Direct comparisons between task and rest revealed significant differences in the VNS group across 0.2–25.0 s (cluster $p = 0.0075$), and in the sham group across 2.1–25.0 s (cluster $p = 0.0185$).

Velocity of pupil dilation (Fig. 6B)

Figure 6B illustrates the velocity of pupil size changes during the first 5 s after onset. During the task period, the taVNS group showed significantly faster pupil dilation than did the sham group at 0.3–0.47 s after onset (cluster $p = 0.039$).

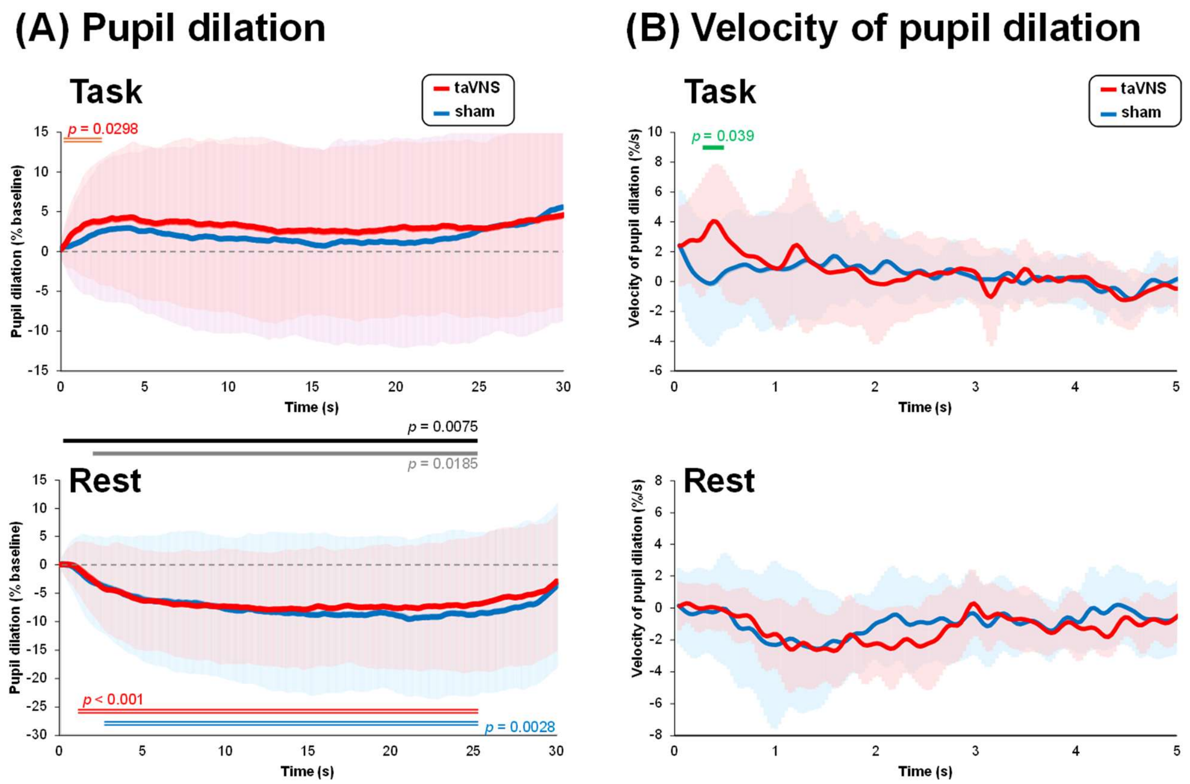

Revised Figure 6A and 6B

Comments to the Author:

Please note that the Journal does not permit the inclusion of substantive content as "Supplementary Information". All relevant information should be included in the main body of the manuscript. As there is no word limit, this requirement should not pose a challenge. The "Supporting Information for online publication" should be used only for "Data set/Video/Audio only".

Response:

We thank the Senior Editor for this guidance. In accordance with the journal policy, we have incorporated the validation experiment previously provided as Supplementary Information into the main manuscript as a new section in the Results. The corresponding figure has also been included in the main figures (please see the lines 471-).

REFEREE COMMENTS

Referee #1:

The authors have suitably addressed most of my original comments. I do, however, have a couple of additional comments based on the responses provided.

Regarding the controlling of light levels within the experimental setting...I cannot see raw data for pupil diameter presented anywhere (unless I have missed it). Simply, showing these values to the similar at baseline (not normalised to 100%) would give more confidence in the rebuttal of my initial comment about light levels impacting pupil diameter, and therefore, more confidence in the between condition results.

Response:
To address the reviewer's concern, we added the absolute baseline pupil diameter values (mm) for both task and rest phases and compared them between groups. No significant difference between the groups was observed in either phase, indicating no baseline difference between conditions that would confound the interpretation of pupil responses.
These changes have been incorporated into the Results section and Figure 6.

Change [lines 299-]:
As a control analysis, non-normalized (absolute) baseline pupil diameter for each phase (task and rest) was compared between the groups using unpaired t-tests to confirm the absence of baseline differences.

Change [lines 453-]:
Absolute baseline pupil diameter (Fig. 6C)
Figure 6C shows baseline pupil diameters for each phase and group. Absolute baseline pupil diameter did not differ between groups during either the task baseline period (taVNS vs sham, $t(27) = 0.529$, $p = 0.601$, $d = 0.200$) or the rest baseline period (taVNS vs sham, $t(27) = 0.475$, $p = 0.639$, $d = 0.180$), indicating no baseline differences between conditions that would confound the interpretation of pupil responses.

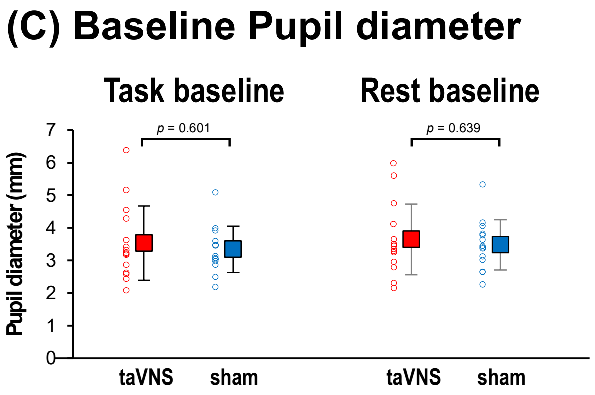

Revised Figure 6C

For MEP and F-wave filtering...I appreciate the additional information although I do wonder why the same filtering was applied for both? Specifically, I understand the high-pass filter setting to help with F-waves (although some studies use even lower), but this seems a little low based on multiple studies of MEP recordings. E.g., Some evidence suggest that high-pass filters set too low can impact measured MEP characteristics: https://pmc.ncbi.nlm.nih.gov/articles/PMC8469458/. Is there any reason why two separate filters were not applied for each measurement. Or additional filtering for F-waves after collection of the initial signals?

Response:

We thank the reviewer for this important comment regarding the filtering of MEP and F-wave signals. We apologise for the insufficient description of our offline filtering procedures in the previous manuscript.

Both MEP and F-wave signals were acquired using the same recording bandwidth (5–1500 Hz). However, different filtering procedures were applied during the analysis stage. Specifically, **an additional high-pass filter at 25 Hz was applied only to the F-wave data**, in line with standard practice (e.g., Kaneko et al. 2022 DOI: 10.1111/ejn.15625), to reduce the influence of the trailing edge of the preceding M-wave and facilitate identification of F-responses.

In contrast, **no additional high-pass filtering was applied to the MEP signals** beyond the acquisition bandwidth. We acknowledge that different high-pass filter settings have been used in the literature for MEP recordings. As evidenced by Zschorlich et al. (2021), higher high-pass filter cutoffs may lead to attenuation of transient MEP signals. In the present study, a 5 Hz cutoff was used, which, while higher than the lowest values recommended in some studies, remains within a commonly used range (e.g., Bjørndal et al. 2024 DOI: 10.1038/s41467-024-49478-5; Horinouchi et al. 2024 DOI: 10.1038/s41598-024-61958-8; Sasaki et al. 2021 DOI: 10.3390/jcm10163633) and is unlikely to substantially distort MEP amplitude measures.

Importantly, identical acquisition and analysis procedures were applied consistently across all participants and experimental conditions. Therefore, any potential influence of filtering would not be expected to systematically bias the between-group or pre–post comparisons.

We have now clarified these points in the revised Methods section.

Change [lines 204-]:

EMG signals were amplified (MEB-6108 amplifier, Nihon Kohden, Tokyo, Japan), band-pass filtered (5–1500 Hz), and digitised at 4000 Hz with an A/D converter (Powerlab, AD Instrument, Sydney, Australia) for both MEP and F-wave recordings.

Change [lines 245-]:

For F-wave analysis, an additional high-pass filter at 25 Hz was applied offline to suppress the trailing edge of the preceding M-wave (Kaneko et al., 2022).

Referee #2:

The authors have significantly enhanced the transparency of the study, and addressed most prior concerns appropriately. It is appreciated that the authors have collected additional data to demonstrate the reliability of the study, and this adds additional support for the methods used. There are some minor comments listed below to further enhance the transparency of the manuscript.

Line 219 to 222: While it is appreciated that the authors have now clarified how the coil position was monitored, there should still be mention of how tilt and orientation was managed and kept consistent (i.e., whether the same or different experimenters performed TMS across different sessions).

Response:

We thank the reviewer for this helpful comment. All TMS measurements were performed by the same experimenter to ensure consistency. We added this statement to the revised manuscript. Coil orientation was kept constant relative to the hotspot, and tilt was manually adjusted to maintain tangential contact with the scalp throughout the experiment.

Change [line 224]:

All TMS measurements were performed by the same experimenter.

Line 560 to 562: the effects of the descending projections on spinal motoneurons is not clear based on this sentence alone, particularly when considering the function of the reticular formation. Could the authors rephrase or add an additional statement to indicate how, or to what extent this may influence spinal motoneuron activity related to motor learning of such voluntary tasks? This will make the physiological underpinning of the possible plasticity effects more apparent to the reader.

Response:

We thank the reviewer for this insightful comment. We agree that our previous description did not sufficiently explain how descending projections from brainstem structures, particularly the reticular formation, may influence spinal motoneuron activity in the context of voluntary motor tasks.

To address this point, we have revised the manuscript to clarify the functional role of these pathways. In addition to their anatomical projections, reticulospinal neurons arising from the pontomedullary reticular formation project to spinal interneurons and motoneurons and are known to contribute to the coordination of muscle activation patterns and to the tuning of descending motor commands during voluntary movement. This suggests that these descending pathways may influence the overall excitability state of the spinal motor system during task execution, thereby indirectly shaping the expression of spinal plasticity.

We have incorporated this clarification in the revised Discussion, which we believe better clarifies the potential physiological mechanisms through which taVNS may influence both cortical and spinal circuits.

Change [lines 596-]:

At the same time, LC neurons send noradrenergic descending projections to the spinal cord, including the ventral horn and intermediate zone, suggesting a potential route by which taVNS-related neuromodulatory signals could influence spinal circuitry (Proudfit and Clark, 1991). In addition, reticulospinal neurons arising from the pontomedullary reticular formation project to spinal interneurons and motoneurons involved in posture and voluntary movement, and are known to contribute to the coordination of muscle activation patterns and to the tuning of descending motor commands during voluntary movement (Brownstone and Chopek, 2018). These pathways may influence the overall excitability state of the spinal motor system during voluntary tasks, thereby indirectly shaping the expression of spinal plasticity. Thus, taVNS may influence the level at which plasticity is expressed within the motor system, spanning both cortical and spinal circuits.

Figure 7: Additional detail should be added to the figure caption to improve the clarity of the proposed plasticity pathway/effects represented in this figure. While the figure itself is well presented, a more detailed description to provide necessary supporting information will improve the interpretability.

Response:

We thank the reviewer for the constructive comment. We have revised the figure caption to provide a more detailed description of the proposed pathways and their potential influence on M1 plasticity, thereby improving the clarity and interpretability of the figure.

Change [lines 663-]:

Figure 8. Putative neural pathways underlying M1 plasticity induced by the combination of motor practice and taVNS

taVNS activates brainstem regions including the nucleus of the solitary tract (NTS), which projects to the locus coeruleus (LC) and basal forebrain (BF), as well as to the cerebellum. These pathways are proposed to influence M1 plasticity via neuromodulatory systems (e.g., noradrenergic and cholinergic projections) and cerebello–thalamo–cortical circuits. Arrows indicate putative pathways through which taVNS may influence cortical plasticity, including both direct projections and neuromodulatory influences.

Abbreviations: BF, basal forebrain; BDNF, brain-derived neurotrophic factor; LC, locus coeruleus; M1, primary motor cortex; NTS, nucleus of the solitary tract; taVNS, transcutaneous auricular vagus nerve stimulation.

END OF COMMENTS

Dear Dr Nakagawa,

Re: JP-RP-2026-290398R2 "**Transcutaneous auricular vagus nerve stimulation during short-term motor practice drives cortical plasticity without behavioural improvement** " by Kento Nakagawa and Rieko Osu

Thank you for submitting your manuscript to The Journal of Physiology. It has been assessed by a Reviewing Editor and by 0 expert referees and we are pleased to tell you that it is acceptable for publication following satisfactory revision.

The referee reports are copied at the end of this email.

Please address all the points raised and incorporate all requested revisions or explain in your Response to Referees why a change has not been made. We hope you will find the comments helpful and that you will be able to return your revised manuscript within 4 weeks. If you require longer than this, please contact journal staff: jp@physoc.org.

Your revised manuscript should be submitted online using the link in your Author Tasks Link Not Available. This link is accessible via your account as Corresponding Author; it is not available to your co-authors. If this presents a problem, please contact journal staff (jp@physoc.org). Image files from the previous version are retained on the system. Please ensure you replace or remove any files that are being revised.

If you do not wish to submit a revised version of your manuscript, you must inform our journal staff (jp@physoc.org) or reply to this email to request withdrawal. Please note that a manuscript must be formally withdrawn from the peer review process at one journal before it may be submitted to another journal.

TRANSPARENT PEER REVIEW POLICY: To improve the transparency of its peer review process The Journal of Physiology publishes online, as supporting information, the peer review history of all articles accepted for publication. Readers will have access to decision letters, including Editors' comments and referee reports, for each version of the manuscript, as well as any author responses to peer review comments. Referees can decide whether or not they wish to be named on the peer review history document.

ABSTRACT FIGURES: Authors are expected to use The Journal's premium BioRender account to create/redraw their Abstract Figures. Information on how to access this account is here: https://physoc.onlinelibrary.wiley.com/journal/14697793/biorender-access.

This will enable Authors to create and download high-resolution figures. If authors have used the free BioRender service, they can use the instructions provided in the link above to download a high-resolution version suitable for publication.

The link provided should only be used for the purposes of this submission. Authors will be charged for figures created on this account if they are not related to this manuscript submission.

LANGUAGE EDITING AND SUPPORT FOR PUBLICATION: If you would like help with English language editing, or other article preparation support, Wiley Editing Services offers expert help, including English Language Editing, as well as translation, manuscript formatting, and figure formatting at www.wileyauthors.com/eeo/preparation. You can also find resources for Preparing Your Article for general guidance about writing and preparing your manuscript at www.wileyauthors.com/eeo/prepresources.

RIGOUR AND REPRODUCIBILITY: We recommend authors consult The Journal's Rigour and Reproducibility requirements to ensure that their manuscript is suitable for publication: https://physoc.onlinelibrary.wiley.com/pb-assets/hub-assets/physoc/documents/TJP-Rigour-and-Reproducibility-Requirements.pdf

REVISION CHECKLIST:

Check that your Methods section conforms to journal policy: https://jp.msubmit.net/cgi-bin/main.plex?form_type=display_requirements#methods

Check that data presented conforms to the statistics policy: https://jp.msubmit.net/cgi-bin/main.plex?form_type=display_requirements#statistics

Upload a full Response to Referees file. To create your 'Response to Referees' copy all the reports, including any comments from the Senior and Reviewing Editors, into a Microsoft Word, or similar, file and respond to each point, using font or background colour to distinguish comments and responses and upload as the required file type.

Please upload two versions of your manuscript text: one with all relevant changes highlighted and one clean version with no changes tracked. The manuscript file should include all tables and figure legends, but each figure/graph should be uploaded as separate, high-resolution files. The journal is now integrated with Wiley's Image Checking service. For further details, see: https://www.wiley.com/en-us/network/publishing/research-publishing/trending-stories/upholding-image-integrity-wileys-image-screening-service

You may also upload:

- 'Potential Cover Art' for consideration as the issue's cover image
- Appropriate Supporting Information (Video, audio or data set: see https://jp.msubmit.net/cgi-bin/main.plex?form_type=display_requirements#supp).

We look forward to receiving your revised submission.

If you have any queries, please reply to this email and we will be pleased to advise.

Yours sincerely,

Richard Carson
Senior Editor
The Journal of Physiology

REQUIRED ITEMS

EDITOR COMMENTS

Reviewing Editor:

The authors have further refined their manuscript in response to comments from the referees and editors, and have done a good job in doing so. Please note that the error bars are not defined for Figure 6C. If these are SD, please indicate this in the figure legend. If these are SEM, please change to SD and indicate this in the figure legend.

Senior Editor:

Please refer to the comments provided by the Reviewing Editor.

END OF COMMENTS

EDITOR COMMENTS

Reviewing Editor:

The authors have further refined their manuscript in response to comments from the referees and editors, and have done a good job in doing so. Please note that the error bars are not defined for Figure 6C. If these are SD, please indicate this in the figure legend. If these are SEM, please change to SD and indicate this in the figure legend.

Senior Editor:

Please refer to the comments provided by the Reviewing Editor.

Response:

We thank the Reviewing Editor and Senior Editor for this comment. We have clarified in the figure legend that the error bars in Figure 6C represent ± SD.

Change [lines 470]: Error bars indicate ± SD.

END OF COMMENTS

Dear Dr Nakagawa,

Re: JP-RP-2026-290398R3 "**Transcutaneous auricular vagus nerve stimulation during short-term motor practice drives cortical plasticity without behavioural improvement** " by Kento Nakagawa and Rieko Osu

We are pleased to tell you that your paper has been accepted for publication in The Journal of Physiology.

The last Word (or similar) version of the manuscript provided will be used by the Production Editor to prepare your proof. When this is ready you will receive an email containing a link to Wiley's Online Proofing System. The proof should be thoroughly checked and corrected as promptly as possible.

Authors should note that it is too late at this point to offer corrections prior to proofreading. Major corrections at proof stage, such as changes to figures, will be referred to the Editors for approval before they can be incorporated. Only minor changes, such as to style and consistency, should be made at proof stage. Changes that need to be made after proof stage will usually require a formal correction notice.

All queries at proof stage should be sent to: TJP@wiley.com.

Following copyediting and typesetting the accepted version will be published online.

Yours sincerely,

Richard Carson
Senior Editor
The Journal of Physiology

IMPORTANT POINTS TO NOTE FOLLOWING ACCEPTANCE OF YOUR PAPER:

• IMPORTANT NOTICE ABOUT OPEN ACCESS: To assist authors whose funding agencies mandate immediate public access to published research findings, The Journal of Physiology allows authors to pay an Open Access (OA) fee to have their papers made freely available immediately on publication.

The Corresponding Author will receive an email from Wiley with details on how to register or log in to Wiley Authors where you will be able to place an order.

• You can check if your funder or institution has a Wiley Open Access Account here:
https://authors.wiley.com/author-resources/Journal-Authors/open-access/author-compliance-tool.html

• TRANSPARENT PEER REVIEW POLICY: To improve the transparency of its peer review process, The Journal of Physiology publishes online as supporting information the peer review history of all articles accepted for publication. Readers will have access to decision letters, including Editors' comments and referee reports, for each version of the manuscript as well as any author responses to peer review comments. Referees can decide whether or not they wish to be named on the peer review history document.

• You can help your research get the attention it deserves! Check out Wiley's free Promotion Guide for best-practice recommendations for promoting your work at: www.wileyauthors.com/eeo/guide. You can learn more about Wiley Editing Services which offers professional video, design, and writing services to create shareable video abstracts, infographics, conference posters, lay summaries, and research news stories for your research at: www.wileyauthors.com/eeo/promotion.

• If you would like to receive our 'Research Roundup', a monthly newsletter highlighting the cutting-edge research published in The Physiological Society's family of journals (The Journal of Physiology, Experimental Physiology, Physiological Reports, The Journal of Nutritional Physiology and The Journal of Precision Medicine: Health and Disease), please click this link, fill in your name and email address and select 'Research Roundup':
https://www.physoc.org/journals-and-media/membernews

EDITOR COMMENTS

Reviewing Editor:

Thank you for addressing all comments from the reviewers and editors.
